# Assessing the impacts of dam/weir operation on streamflow predictions using LSTM across South Korea

**DOI:** 10.1038/s41598-023-36439-z

**Published:** 2023-06-08

**Authors:** Yongsung Kwon, YoonKyung Cha, Yeonjeong Park, Sangchul Lee

**Affiliations:** 1 Department of Environmental Engineering, University of Seoul, Dongdaemun-gu, Seoul, 02504 South Korea; 2 Water Quality Assessment Research Division, Water Environment Research Department, National Institute of Environmental Research, Incheon, 22689 South Korea

**Keywords:** Environmental sciences, Hydrology

## Abstract

Recently, weather data have been applied to one of deep learning techniques known as “long short-term memory (LSTM)” to predict streamflow in rainfall-runoff relationships. However, this approach may not be suitable for regions with artificial water management structures such as dams and weirs. Therefore, this study aims to evaluate the prediction accuracy of LSTM for streamflow depending on the availability of dam/weir operational data across South Korea. Four scenarios were prepared for 25 streamflow stations. Scenarios #1 and #2 used weather data and weather and dam/weir operational data, respectively, with the same LSTM model conditions for all stations. Scenarios #3 and #4 used weather data and weather and dam/weir operational data, respectively, with the different LSTM models for individual stations. The Nash–Sutcliffe efficiency (NSE) and the root mean squared error (RMSE) were adopted to assess the LSTM’s performance. The results indicated that the mean values of NSE and RMSE were 0.277 and 292.6 (Scenario #1), 0.482 and 214.3 (Scenario #2), 0.410 and 260.7 (Scenario #3), and 0.592 and 181.1 (Scenario #4), respectively. Overall, the model performance was improved by the addition of dam/weir operational data, with an increase in NSE values of 0.182–0.206 and a decrease in RMSE values of 78.2–79.6. Surprisingly, the degree of performance improvement varied according to the operational characteristics of the dam/weir, and the performance tended to increase when the dam/weir with high frequency and great amount of water discharge was included. Our findings showed that the overall LSTM prediction of streamflow was improved by the inclusion of dam/weir operational data. When using dam/weir operational data to predict streamflow using LSTM, understanding of their operational characteristics is important to obtain reliable streamflow predictions.

## Introduction

Advanced monitoring networks have led to the automatic collection of large-scale data, and enhanced computing resources and algorithms have efficiently analyzed these datasets^1^. Deep learning is a crucial computing algorithm for data analysis and is widely used in various fields owing to its superior prediction and classification abilities^2^. The key deep learning techniques frequently used are recurrent neural networks (RNN) for sequence data and convolutional neural networks (CNN) for images^3^. These advanced deep-learning techniques have also been adopted in environmental fields, such as disaster management, water resource management systems, and air pollution problems^4^. Water resource data are regularly collected, and these sequence data are recorded at successive equally spaced points in time. Therefore, RNN models are frequently used in water resources^5^. Therefore, hydrological variables, such as rainfall, outflow, water demand, and water level, are often predicted using RNN models^6^.

Recently, weather data have been used to predict streamflow using RNN. The most frequently used RNN models are updated versions of the original RNN^7^, including long short-term memory (LSTM)^8^ and gated recurrent unit (GRU)^9^, which address the vanishing gradient problem of the original RNN^10^. Fu et al.^11^ used the LSTM model to predict the streamflow of the Kelantan River in northeastern Malaysia over the past 50 years using rainfall data. Rahimzad et al.^12^ used rainfall data to predict streamflow using LSTM. They found that LSTM outperformed machine-learning models (e.g., linear regression, multilayer perceptron, and support vector machines). Wang et al.^13^ collected streamflow and rainfall data from seven watersheds in China and predicted streamflow using a GRU model. A hybrid approach has been suggested to improve LSTM and GRU's prediction capacity. Masrur Ahmed et al.^15^ coupled LSTM and GRU with the Boruta feature selection algorithm (BRF) to predict streamflow using weather data and developed a principal component analysis (PCA), LSTM, and Bayesian optimization (BO) that decomposes and ensembles to improve the accuracy of predicting the runoff of the Huangshui River in eastern Qinghai Province, China^14^.

Regarding rainfall-runoff relationships, deep learning models have frequently adopted weather data as input data for predicting streamflow^16^. However, this common practice may be unsuitable for stream networks with abundant artificial construction, such as dams and weirs, because streamflow is highly affected by their operations^17^. In addition, implementing artificial construction modifies streamflow patterns regardless of the weather conditions^18^. Therefore, including such operational data could improve the prediction accuracy of deep learning models for streamflow. The study by Ouyang et al.^19^ compared the performance of the LSTM model depending on the degree of dam influence for 3557 basins across the United States, emphasizing the importance of dams being treated appropriately in the LSTM model when forecasting streamflow.

This study aims to quantitatively assess the impact of dam/weir operational data on the capacity of LSTM to predict streamflow in South Korea. Dams and weirs have been intensively constructed for water resource management in South Korea^20^. Due to the prevalence of mountainous areas and concentrated precipitation^21^, securing water resources is critical in South Korea. Efficient management of water resources in South Korea and the Four River Restoration Project between 2009 and 2012 led to the implementation of dams and weirs in a 537-km river channel^21^. Thus, South Korea is a suitable test bed to test the impacts of dam/weir operational data on deep learning models regarding geographical and climatic conditions. Furthermore, extensive dam/weir constructions were made for a short period in South Korea and thus our study is helpful for the regions that experience rapid development of water management constructions.

To demonstrate the importance of dam/weir operational data for the LSTM model, we first identified 25 streamflow stations based on the data availability. Then, we have prepared four scenarios with different input data and optimization conditions. The first two scenarios were set to have the same optimal hyperparameters for all 25 streamflow stations while one scenario only had climatic input data (Scenario #1) and the other one used climatic as well as dam/weir operational data (Scenario #2). The other two scenarios were set the have different optimal hyperparameters for 25 individual streamflow stations while one scenario only had climatic input data (Scenario #3) and the other one included climatic as well as dam/weir operational data (Scenario #4). By comparing four scenarios, this study explored the impact of dam/weir operational data on the capacity of LSTM to predict streamflow in South Korea.

## Materials and methods

### Study area

**Figure 1:**
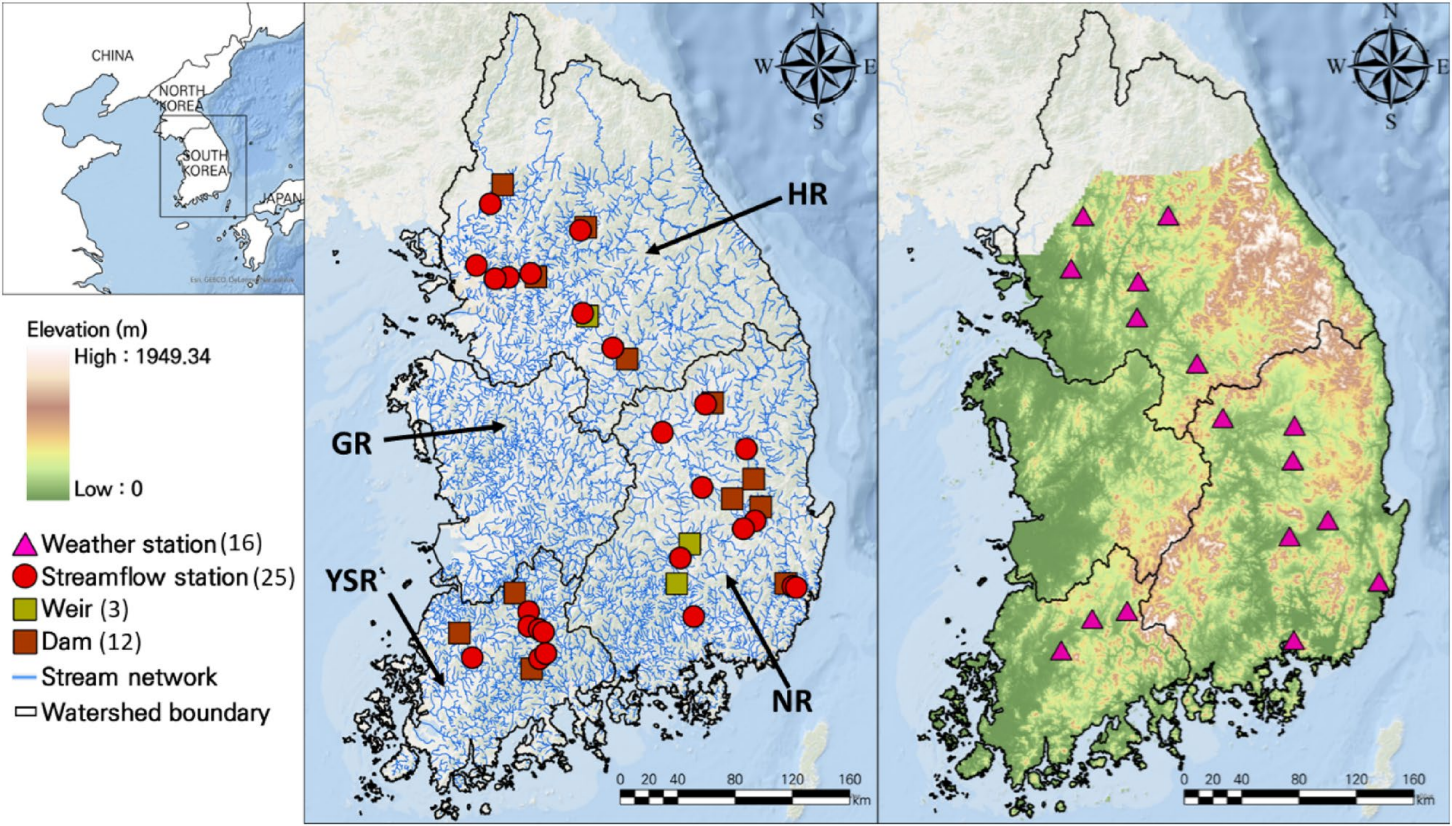
The location of the four watersheds, weather stations, streamflow stations, weirs, and dams. The number within the parenthesis on the legend indicates the number of stations. HR, GR, NR, and YSR represent the Han River, Geum River, Nakdong River, and Youngsan/Seomjin River, respectively.

South Korea is located at 127° east longitude and 37° north latitude (Fig. 1)^22^. Geographically, it is an East Asian region heavily influenced by the Asian monsoons and has four distinct seasons^23^. The average annual temperature ranges from 3.1 to 13.2 °C and varies by geographical location (e.g., distances to oceans, elevations, etc.)^24^. The winter (December to February) and spring seasons (March–May) tend to be dry due to the northwest seasonal winds caused by the Siberian high pressure^25–28^. Moreover, the East Asian region is prone to be humid during the summer season (June to August) due to the influence of the North Pacific high-pressure system^29^. Over 50% of the average annual precipitation is concentrated in the summer^30^, making it important to carefully manage water resources. South Korea has implemented Integrated Water Resources Management (IWRM) policies to efficiently manage water resources within the four major watersheds, which are managed separately for efficient water management^31^.

### Data acquisition

This study used daily streamflow, weather, and dam/weir operation data between 2010 and 2020 (Table 1). The temporal coverage was determined based on the availability of the three datasets. Daily streamflow data were downloaded from the Water Resources Management Information System (WAMIS, http://wamis.go.kr/). The 25 streamflow stations were chosen based on data availability and the spatial locations of the dams and weirs. Daily weather data (e.g., precipitation, maximum and minimum temperatures, and relative humidity) were obtained from 16 automated synoptic observing systems (ASOS) operated by the Korea Meteorological Administration (KMA, https://data.kma.go.kr/). The weather stations closest to the individual stream stations were selected. The twelve dams and three weirs adjacent to 25 streamflow stations were used. Their daily inflow and outflow data were obtained from WAMIS and the Korea Water Resources Corporation (Mywater, https://www.water.or.kr/). There were no suitable streamflow stations in the GR due to insufficient weather and dam/weir operational data.

**Table 1:** Descriptions of input variables.

Variable	Description (unit)	Source	Period	Number
Streamflow	Daily streamflow (m^3^/s)	Mywater	2010.01.01–2020.12.31	25
Weather	Daily precipitation (mm/s) Maximum temperature (°C) Minimum temperature (°C) Relative humidity (%)	KMA	2010.01.01–2020.12.31	16
Dam*	Daily inflow, outflow (m^3^/s)	WAMIS Mywater	~ 2020.12.31	12
Weir*	Daily inflow, outflow (m^3^/s)	WAMIS Mywater	~ 2020.12.31	3

*KMA* Korea Meteorological Administration, *WAMIS* water resources management information system.

*The detailed information of weather, streamflow, and dam/weir is summarized in Tables S1, S2, and S3 of the Supplementary Material, respectively.

### Study design

**Figure 2:**
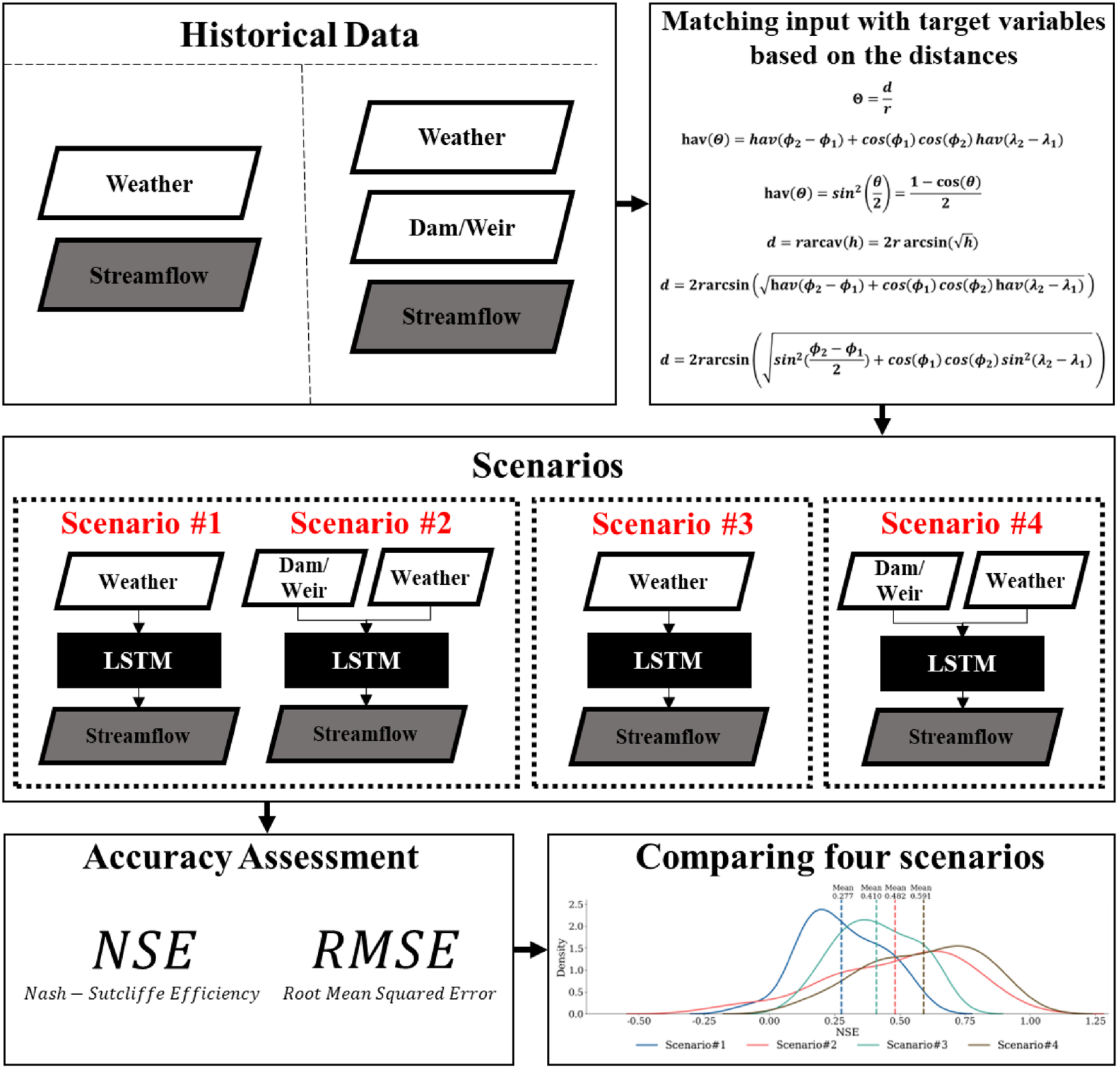
Schematic diagram of this study.

To demonstrate the importance of dam/weir operational data in predicting streamflow using LSTM in South Korea, this study developed four scenarios with different input data and different hyperparameter optimization techniques (Fig. 2). Scenarios #1 and #2 have varying input data (one with weather [Scenario #1] data and the other with weather and dam/weir operational data [Scenario #2]). The two LSTM models were optimized to have the same hyperparameter values for all 25 streamflow stations. Scenarios #1 and #2 were intended to determine how the LSTM performed when all conditions were the same except for the input data. Scenarios #3 and #4 had different hyperparameter values of LSTM models for individual 25 streamflow stations, but Scenario #3 had only weather data as LSTM input data. In contrast, Scenario #4 used weather and dam/weir operational data for LSTM. Scenarios #3 and #4 were optimized using Bayesian optimization. Using four scenarios, this study aimed to demonstrate the importance of dam/weir operational data for streamflow predictions under different LSTM conditions. The analysis procedure included data preprocessing, matching the input with target variables, the LSTM model, hyperparameter optimization, and objective function (Fig. 2).

### Data preprocessing

The treatment of missing values for each variable was conducted differently by referring to previous studies^32,33^. First, missing precipitation values were replaced with zeros. Second, previous one-day observations were used to fill in the missing values for maximum and minimum temperatures, and relative humidity^34^. Third, missing values for the dam/weir operational data were predicted using linear interpolation^35^. Finally, after processing the missing values for each input data point, all input data were normalized to values between 0 and 1.

$${\mathrm{Y}}_{\mathrm{i}}=\frac{{\mathrm{X}}_{\mathrm{i}}-{\mathrm{X}}_{\mathrm{min}}}{{\mathrm{X}}_{\mathrm{max}}-{\mathrm{X}}_{\mathrm{min}}}$$

where ${\mathrm{Y}}_{\mathrm{i}}$ is the value of a normalized variable, and ${\mathrm{X}}_{\mathrm{i}}, {\mathrm{X}}_{\mathrm{min}},\mathrm{ and }{\mathrm{X}}_{\mathrm{max}}$ represent the observed, maximum, and minimum values of the observation, respectively.

### Matching input with target variables

The weather stations closest to the individual 25 streamflow monitoring stations were identified using a Haversine function that computes the closest distance between the two points to match the input and target data acquired from numerous monitoring stations. This computation was performed using the Python library (haversine). Consequently, the 16 weather monitoring stations were matched to 25 streamflow stations (Fig. 1b). Regarding the flow direction and location, 12 dams and three weirs upstream of 25 streamflow stations were selected (Fig. 1a). The weather stations and dam/weir matched with streamflow stations were summarized in Table S4 of the Supplementary Material.

### Long short‑term memory

The LSTM is a more advanced version of the RNN that has gradient vanishing and exploding problems as data size increases^36^. To overcome the RNN problem, the LSTM has two states (cell and hidden states) with three gates (forget, input, and output) to determine which data to forget, store, and read, and uses three gates and two states^12^. For example, the forget gate (${\mathrm{f}}_{\mathrm{t}}$) determines how much it will forget the past information from the previous cell state (${\mathrm{C}}_{\mathrm{t}-1}$) by passing the previous hidden state (${\mathrm{h}}_{\mathrm{t}-1}$) and current input data (${\mathrm{x}}_{\mathrm{t}}$) through the sigmoid function ($\upsigma$).

$${\mathrm{f}}_{\mathrm{t}}=\upsigma ({\mathrm{W}}_{\mathrm{f},\mathrm{x}}{\mathrm{x}}_{\mathrm{t}}+{\mathrm{W}}_{\mathrm{f},\mathrm{h}}{\mathrm{h}}_{\mathrm{t}-1}+{\mathrm{b}}_{\mathrm{f}})$$

where ${\mathrm{W}}_{\mathrm{f},\mathrm{x}}$ and ${\mathrm{W}}_{\mathrm{f},\mathrm{h}}$ denote the weights linking the forget gate with the ${\mathrm{x}}_{\mathrm{t}}$ and ${\mathrm{h}}_{\mathrm{t}-1}$; ${\mathrm{b}}_{\mathrm{f}}$ denotes the bias vector of the forget gate.

The next step was to add new information to the cell state. This process consisted of two parts. The first part is the input gate (${\mathrm{i}}_{\mathrm{t}}$), which uses a sigmoid function to determine the value to update. The second part is a hyperbolic tangent (tanh) layer that generates a vector of new candidate values that can be added to the cell state. The sigmoid function (σ) is calculated by adjusting the weights of the previous hidden state (${\mathrm{h}}_{\mathrm{t}-1}$) and the current input data (${\mathrm{x}}_{\mathrm{t}}$). Similarly, the hyperbolic tangent (tanh) generating the candidate cell (${\widetilde{\mathrm{C}}}_{\mathrm{t}}$) used to update the new cell is calculated by adjusting the weight of the previous hidden state (${\mathrm{h}}_{\mathrm{t}-1}$) and the current input (${\mathrm{x}}_{\mathrm{t}}$).

$${\mathrm{i}}_{\mathrm{t}}=\upsigma ({\mathrm{W}}_{\mathrm{i},\mathrm{x}}{\mathrm{x}}_{\mathrm{t}}+{\mathrm{W}}_{\mathrm{i},\mathrm{h}}{\mathrm{h}}_{\mathrm{t}-1}+{\mathrm{b}}_{\mathrm{i}})$$

$${\widetilde{\mathrm{C}}}_{\mathrm{t}}=\mathrm{tanh}({\mathrm{W}}_{\mathrm{C},\mathrm{x}}{\mathrm{x}}_{\mathrm{t}}+{\mathrm{W}}_{\mathrm{C},\mathrm{h}}{\mathrm{h}}_{\mathrm{t}-1}+{\mathrm{b}}_{\mathrm{C}})$$

where ${\mathrm{W}}_{\mathrm{i},\mathrm{x}}$ and ${\mathrm{W}}_{\mathrm{i},\mathrm{h}}$ represent the weights connecting the input gate to the ${\mathrm{x}}_{\mathrm{t}}$ and ${\mathrm{h}}_{\mathrm{t}-1}$; ${\mathrm{b}}_{\mathrm{i}}$ denote bias vectors of the input gate, respectively; ${\mathrm{W}}_{\mathrm{C},\mathrm{x}}$ and ${\mathrm{W}}_{\mathrm{C},\mathrm{h}}$ represent the weights connecting the candidate cell to the ${\mathrm{x}}_{\mathrm{t}}$ and ${\mathrm{h}}_{\mathrm{t}-1}$; ${\mathrm{b}}_{\mathrm{C}}$ denotes the bias vectors of the candidate cell. In the next step, the current cell state (${\mathrm{C}}_{\mathrm{t}}$) is updated by combining the previous cell state (${\mathrm{C}}_{\mathrm{t}-1}$) and the candidate cell (${\widetilde{\mathrm{C}}}_{\mathrm{t}}$).

$${\mathrm{C}}_{\mathrm{t}}={\mathrm{f}}_{\mathrm{t}}{\mathrm{C}}_{\mathrm{t}-1}+ {\mathrm{i}}_{\mathrm{t}}{\widetilde{\mathrm{C}}}_{\mathrm{t}}$$

**Figure 3:**
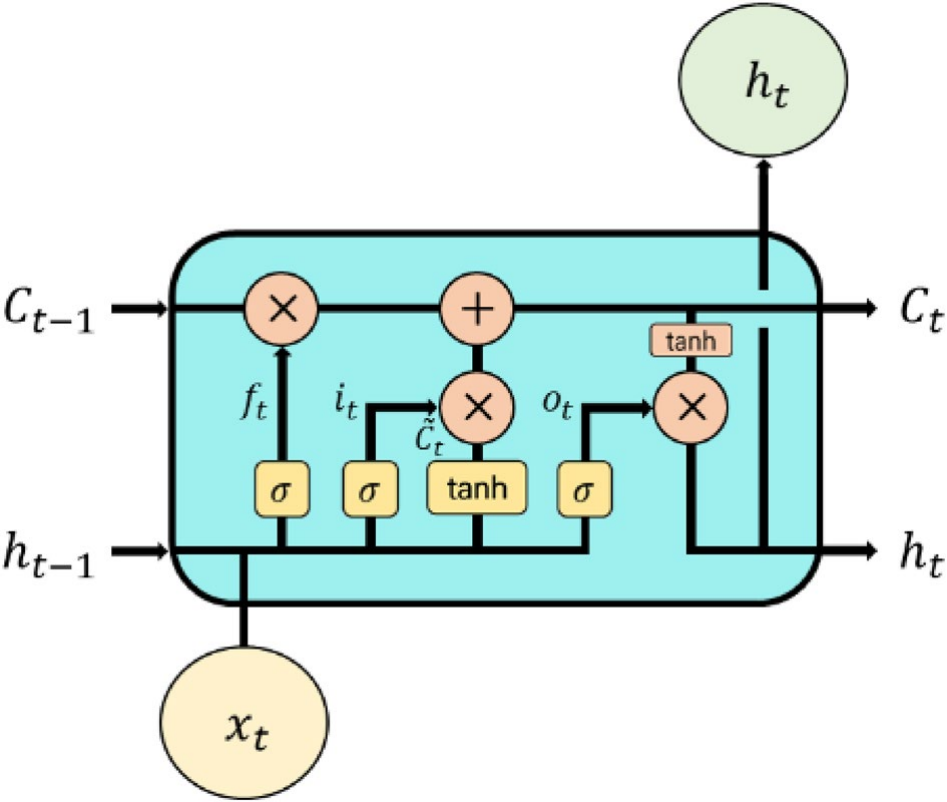
A diagram of long short-term memory structure (adopted from Rahimzad et al.^12^).

The output gate (${\mathrm{o}}_{\mathrm{t}}$) determines the amount of output to be exported from the cell using the sigmoid function through a weight adjustment of the previous hidden state (${\mathrm{h}}_{\mathrm{t}-1}$) and the current input data (${\mathrm{x}}_{\mathrm{t}}$). The hyperbolic tangent function can solve the problems of vanishing and exploding gradients while updating a specific point state (${\mathrm{h}}_{\mathrm{t}}$).

$${\mathrm{o}}_{\mathrm{t}}=\upsigma ({\mathrm{W}}_{\mathrm{o},\mathrm{x}}{\mathrm{x}}_{\mathrm{t}}+{\mathrm{W}}_{\mathrm{o},\mathrm{h}}{\mathrm{h}}_{\mathrm{t}-1}+{\mathrm{b}}_{\mathrm{o}})$$

$${\mathrm{h}}_{\mathrm{t}}={\mathrm{o}}_{\mathrm{t}}\times \mathrm{tanh}({\mathrm{C}}_{\mathrm{t}})$$

where ${\mathrm{W}}_{\mathrm{o},\mathrm{x}}$, ${\mathrm{W}}_{\mathrm{o},\mathrm{h}}$ represent the weights connecting the output gate to the ${\mathrm{x}}_{\mathrm{t}}$, ${\mathrm{h}}_{\mathrm{t}-1}$, and ${\mathrm{b}}_{\mathrm{o}}$ denotes the bias vectors of the output gate (Fig. 3).

The LSTM model has different structures, such as a single LSTM layer, stacked LSTM layers, and bidirectional LSTM^37,38^. This study used two stacked LSTM layers for the four scenarios because a deeper structure provides a more stable prediction capacity for sequence data^39,40^.

### Tuning hyperparameters

Deep learning models were trained to find the models with the best performance by tuning the hyperparameters^41^. The hyperparameters of LSTM include the sequence length, number of hidden layers, number of nodes, number of epochs, dropout rate, learning rate, and batch size. The sequence length determines how much time in the past is used to learn data at any point in time; a node plays an important role in distinguishing the characteristics of input patterns; and the dropout rate prevents overfitting by randomly excluding some of the entire nodes when learning. The learning rate determines how much to learn at once, the batch size means the data size at a time, and the epoch refers to the number of times to learn all training datasets^42^.

The four scenarios had different hyperparameter optimizations to achieve the goals of this study. Following previous studies^19,43^, Scenarios #1 and #2 with different input data (Scenario #1: weather and Scenario #2: weather and dam/weir) were forced to have the same hyperparameter values for all 25 streamflow stations by manual optimization. The optimal hyperparameter values of Scenarios #1 and #2 are shown in Table S5 of the Supplementary Material. Scenarios #3 and #4 were set to have different input data (Scenario #3: weather and Scenario #4: weather and dam/weir) with different hyperparameter values for 25 individual streamflow stations using Bayesian optimization. The values and their ranges of hyperparameters used for Bayesian optimization are shown in Table S6 of the Supplementary Material. The optimal hyperparameter values for Scenarios #3 and #4 were reported in Tables S7 and S8 of the Supplementary Material. Bayesian optimization aims to find the optimal input value that maximizes the objective function *f(x)* that receives the input value *x*. Therefore, the input variable, called the hyperparameter, is modified and set to determine the maximum objective function *f(x)* as follows^44^:

$${x}^{*}= argmax f\left(x\right) (x \in X )$$

where x* is the value of the optimal hyperparameter obtained through Bayesian optimization, argmax *f(x)* is the value of *x* that maximizes *f*, and *x* is the individual parameter for all types of parameter *X*.

Bayesian optimization consists of surrogate models and acquisition functions^45^. The surrogate model approximately estimates the value of *f(x)* based on the results inferred through the Gaussian process. The acquisition function then stochastically proposes the following optimal estimation points based on the estimated mean value and variance range of the uncertainty of the Gaussian process. The purpose of this process is to determine the optimal value by repeating the process of updating the results of estimating the objective function^46^.

### Objective function

This study used the Nash–Sutcliffe efficiency (NSE) to assess the prediction accuracy of the two LSTM models. The NSE has been frequently adopted to quantify the prediction capacity of deep learning models to forecast streamflow^47,48^. The NSE ranges from -∞ to 1, as the NSE approaches 1 (or -∞), simulations agree (or disagree) with observations^49^. The root mean squared error (RMSE) is another commonly used metric to evaluate the accuracy of predicted values. It measures the average squared difference between the predicted and observed values, with a lower RMSE indicating better model performance. Here, we selected the optimal model based on only the NSE values. The performance differences of the optimal LSTM models among four scenarios were compared using both the NSE and RMSE.

$$NSE=1-\frac{\sum_{i=1}^{n}{({O}_{i}-{P}_{i})}^{2}}{\sum_{i=1}^{n}{({O}_{i}-{\overline{O} }_{i})}^{2}}$$

$$RMSE=\sqrt{\frac{{\sum }_{i=1}^{n}{({P}_{i}-{O}_{i})}^{2}}{n}}$$

where ${O}_{i}$ and ${P}_{i}$ denotes the *i*th observed and simulated values, respectively. ${\overline{O} }_{i}$ denotes the average of all the observed values.

## Results and discussions

### Performance comparisons of four scenarios

**Figure 4:**
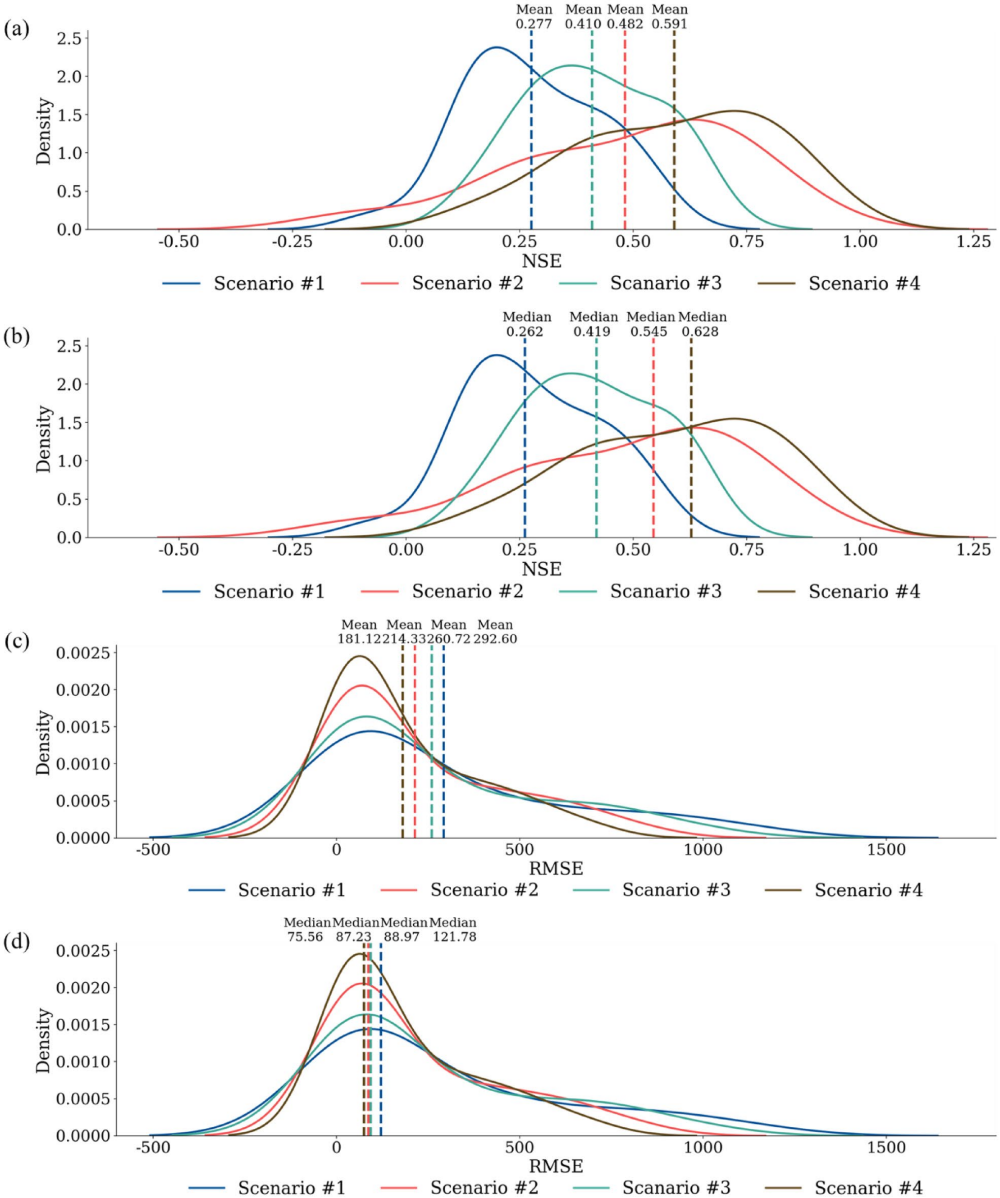
The density plot of NSE and RMSE values with (**a**) mean NSE values, (**b**) median NSE values, (**c**) mean RMSE values, and (**d**) median RMSE values for scenario #1, scenario #2, scenario #3, and scenario #4. The NSE and RMSE values during the train and test periods for all scenarios are shown in Table S9 of the Supplementary Material.

The LSTM prediction results for 25 streamflow stations under the four scenarios were compared using density plots (Fig. 4). The NSE range of Scenario #1 was from − 0.064 to 0.539 with the most right-skewed distribution among the scenarios, and the corresponding range of the RMSE values was from 13.66 to 1118.05. The average and median NSE values were the lowest. Scenario #2 indicated the NSE range from − 0.134 to 0.868 with a left-skewed distribution compared to Scenario #1, exhibiting better prediction accuracy, and its range of the RMSE values was from 12.89 to 800.37. Scenario #3 showed an NSE ranging from 0.128 to 0.658, with a corresponding range of the RMSE values was between 11.68 and 977.27. In contrast, the NSE values shown in Scenario #4 were between 0.160 and 0.901, with the most left-skewed result among all scenarios, and its range of the RMSE values was from 11.59 to 677.67. The mean NSE values between Scenarios #1 and #2 increased 0.277 from 0.482 while the values between Scenarios #3 and #4 increased from 0.410 to 0.591. The reduction value of the mean RMSE was 78.3 between Scenarios #1 and #2, and 79.6 between Scenarios #3 and #4). Overall, the changes in NSE and RMSE results supported the importance of dam/weir operational data in enhancing streamflow predictions.

**Figure 5:**
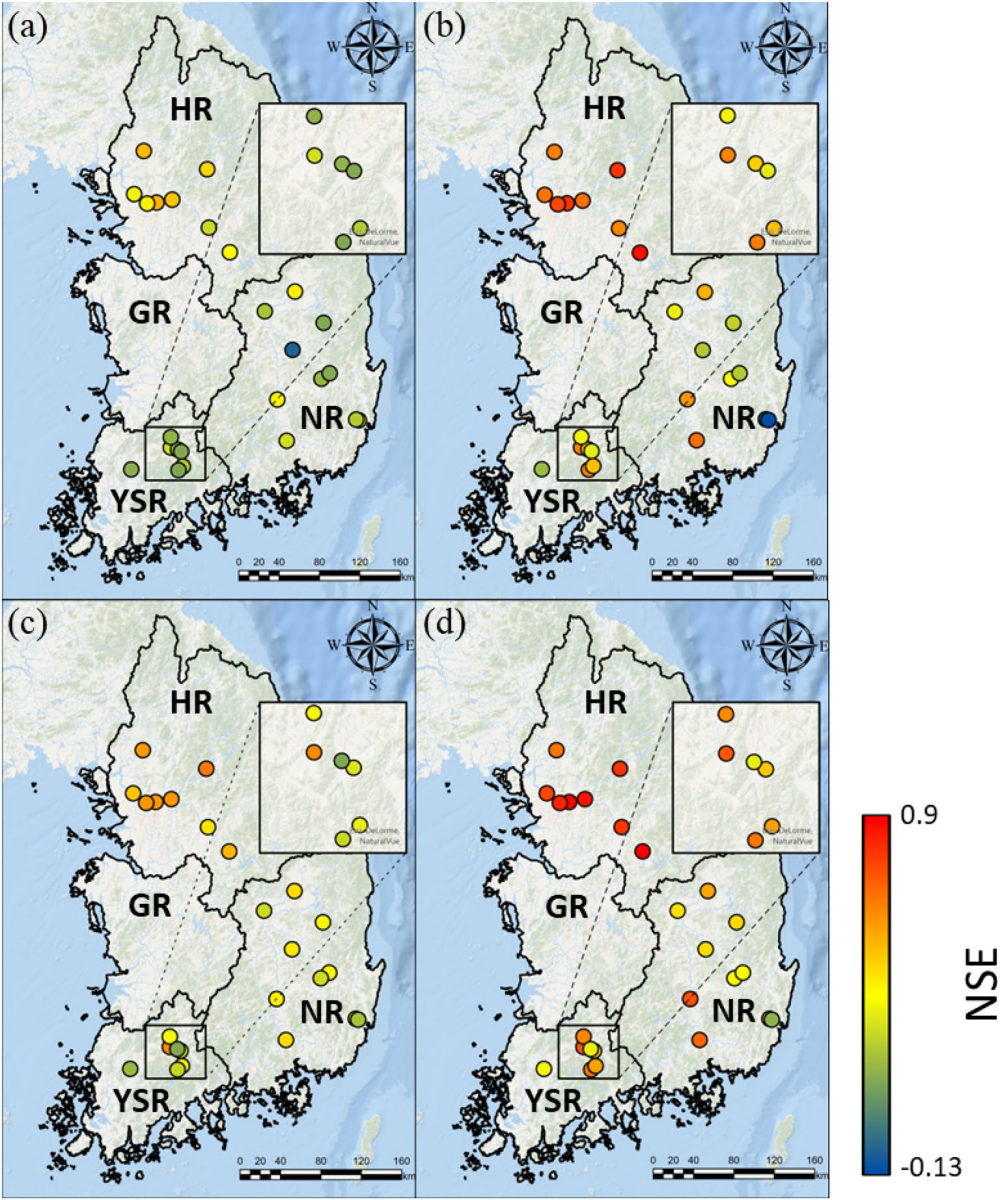
The spatial distribution of NSE values for 25 stations: (**a**) scenario #1, (**b**) scenario #2, (**c**) scenario #3, and (**d**) scenario #4.

**Figure 6:**
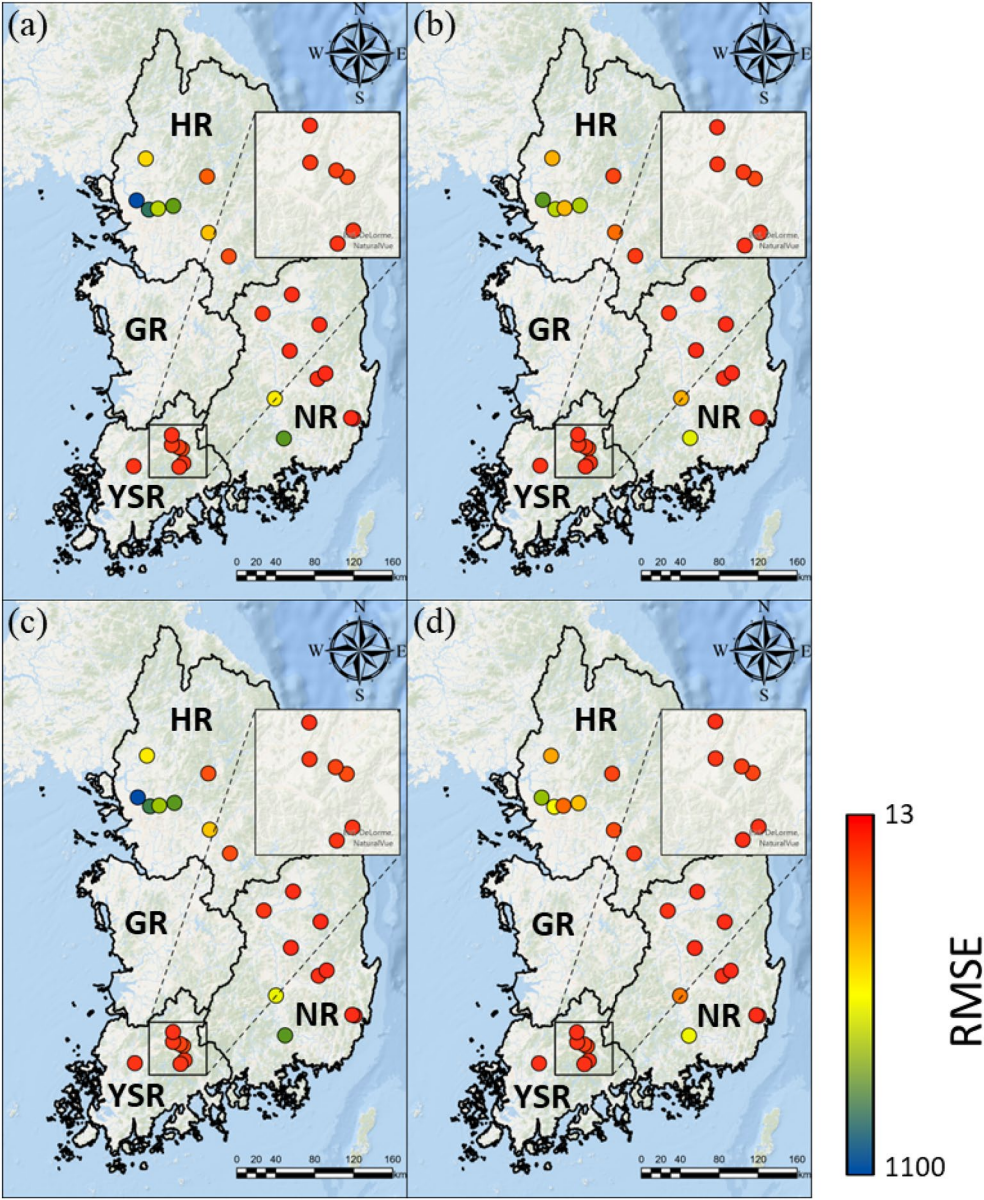
The spatial distribution of RMSE values for 25 stations: (**a**) scenario #1, (**b**) scenario #2, (**c**) scenario #3, and (**d**) scenario #4.

The spatial patterns of the LSTM performance indicated by the NSE and RMSE under the four scenarios are shown in Figs. 5 and 6, respectively. In compliance with Fig. 4, LSTM results with the consideration of the dam/weir operational data (Fig. 5b,d) outperformed in predicting streamflow, without the consideration of these data (Fig. 5a,c). According to the NSE and RMSE results, when the dam/weir operational data were included, the LSTM performance for the HR basin was improved by 0.297 (from Scenario #1 to Scenario #2) and 0.249 (from Scenario #3 to Scenario #4), with the corresponding reduction in RMSE of 180.96 and 189.44, respectively. The YSR showed similar results, with an increased in NSE values of 0.270 (from Scenario #1 to Scenario #2) and 0.225 (from Scenario #3 to Scenario #4), and the reduction in RMSE values of 21.16 and 17.10, respectively. In addition, small increases in NSE values of 0.087 (from Scenario #1 to Scenario #2) and 0.099 (from Scenario #3 to Scenario #4) were observed in the NR basin, along with the reduction in RMSE of 36.11 and 35.48, respectively.

Like our findings, previous studies have reported the enhancement of deep learning model predictions by adding input data. Kim and Kang^50^ developed an LSTM-based daily streamflow estimation model using weather data to predict streamflow in the Soyang River Basin in South Korea, and they reported that the NSE was 0.8 for the model with only precipitation input data while the LSTM performance increased by 0.05 with the inclusion of additional data (e.g., temperature, wind speed, and precipitation). Ouyang et al.^19^ investigated the effects of reservoir data on streamflow prediction using LSTM and found that when reservoir data were included, the NSE values were improved from 0.65 to 0.75. To improve the accuracy of river flow prediction in the snow-covered basin of Kalixälven in northern Sweden, Achite et al.^51^ utilized a snow-based conceptual hydrological model (MISD) and a deep learning model (the group method of data handling, GMDH), and found that the improvement in the model performances by the addition of weather data. A study by Moosavi et al.^52^ evaluated factors affecting the accuracy of daily runoff predictions using various data-driven models and the input data had the most significant impact on the accuracy of model prediction. Based on previous studies, it could be concluded that additional data are important to improve the LSTM prediction accuracy.

### Analysis of streamflow stations with reduced and improved LSTM performance

Including dam/weir operational data did not always improve the LSTM’s performance for all streamflow stations. At the streamflow station (2201660) downstream of the Sayeon Dam in the NR basin (Fig. S1 of the Supplementary Material), the NSE performance decreased by − 0.396 (from Scenario #1 to Scenario #2) and − 0.052 (from Scenario #3 to Scenario #4). This could be explained by the operational characteristics of the Sayeon Dam, which was built as a water supply dam for domestic and industrial use and has a minimal impact on downstream owing to its infrequent water discharge downstream^53^. Furthermore, the East Sea is located 10 km east of the streamflow station, and the difference in height between high and low tides could affect the streamflow^54^. Therefore, these two factors collectively led to a reduction in the LSTM model’s performance with the addition of dam data.

The most significant improvement in dam/weir operational data was observed at the streamflow station (4008660), which is downstream of the Juam multipurpose dam in the YSR basin (Fig. S2 of Supplementary Material). The NSE performance was 0.129 for Scenario #1, 0.650 for Scenario #2, 0.266 for Scenario #3, and 0.661 for Scenario #4. Therefore, when considering the Juam Dam data, the LSTM performance increased by 0.521 (from Scenario #1 to Scenario #2) and 0.395 (from Scenario #3 to Scenario #4). This result disagreed with the streamflow station (2201660) downstream area of Sayeon Dam in the NR basin described above. In general, the discharge amount of a dam is calculated by adding various water supply quantities such as domestic, industrial, agricultural, and power-generation water. Unlike the Juam multipurpose dam with high frequency of water discharge, the Sayeon dam is primarily used for water supply and discharges water intermittently during the flood season.

**Figure 7:**
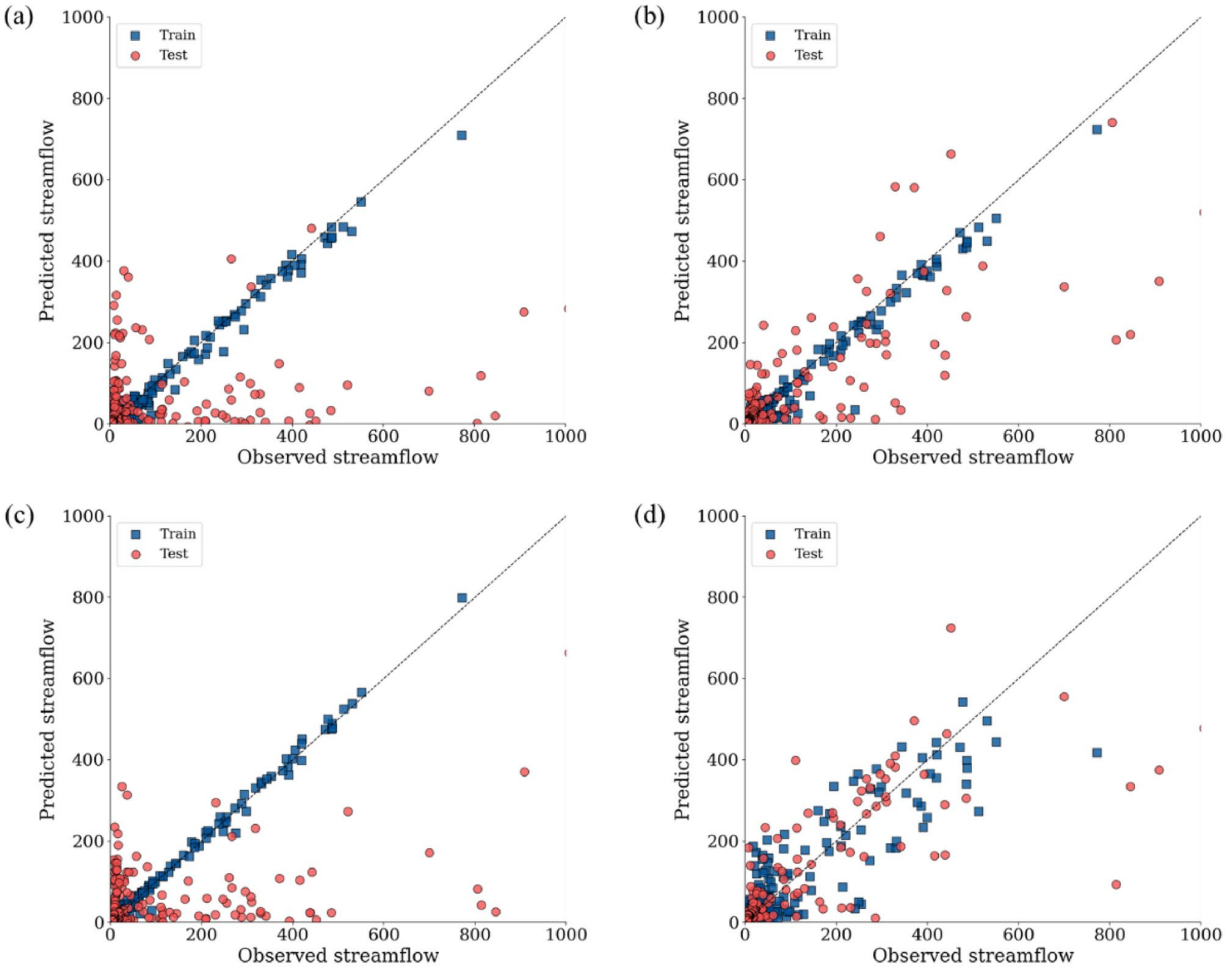
Scatterplots between observed and predicted streamflow for (**a**) scenario #1, (**b**) scenario #2, (**c**) scenario #3, and (**d**) scenario #4 at the streamflow station (4008660).

**Figure 8:**
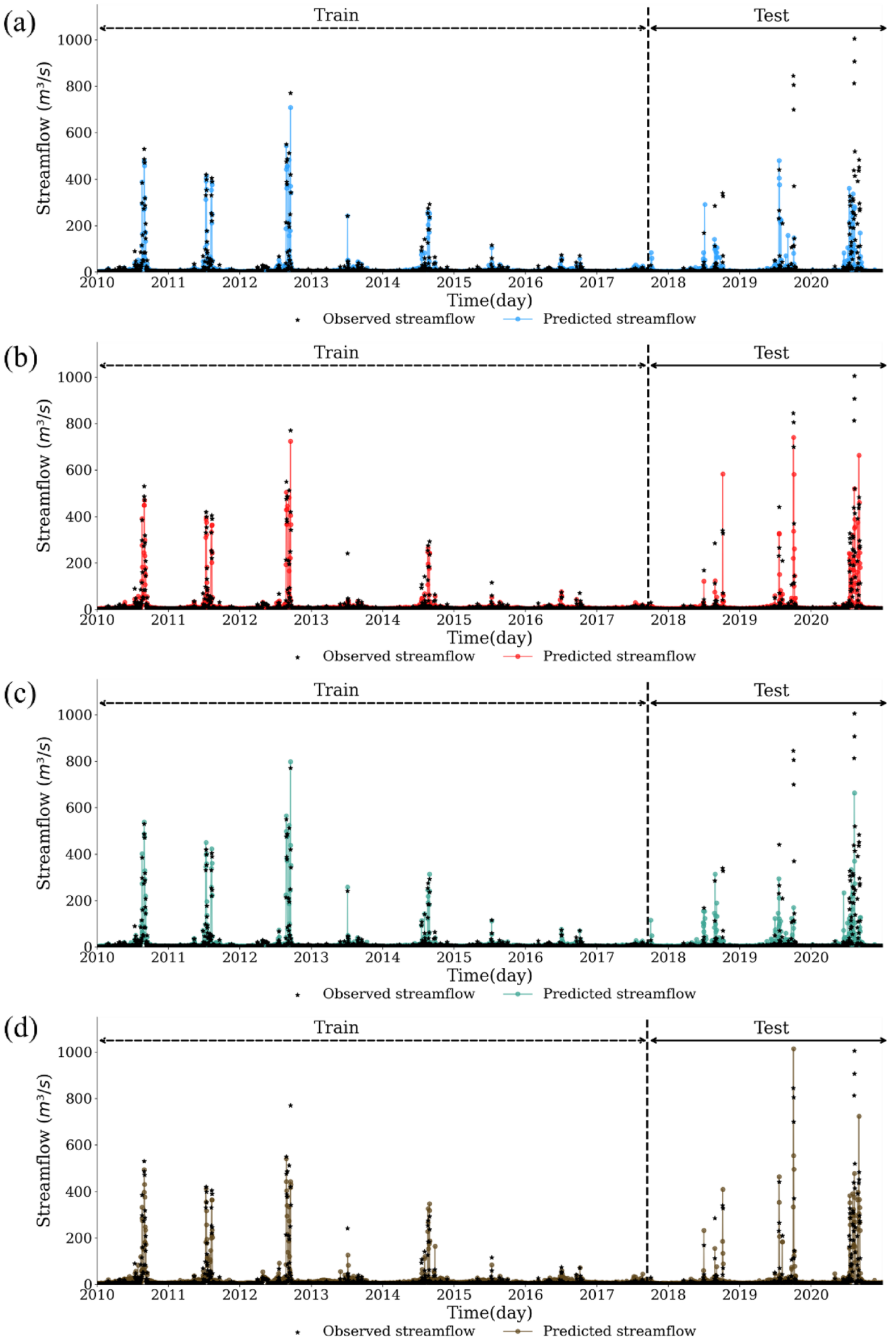
Comparisons between observed and predicted streamflow for (**a**) scenario #1, (**b**) scenario #2, (**c**) scenario #3, and (**d**) scenario #4 at the streamflow station (4008660).

**Figure 9:**
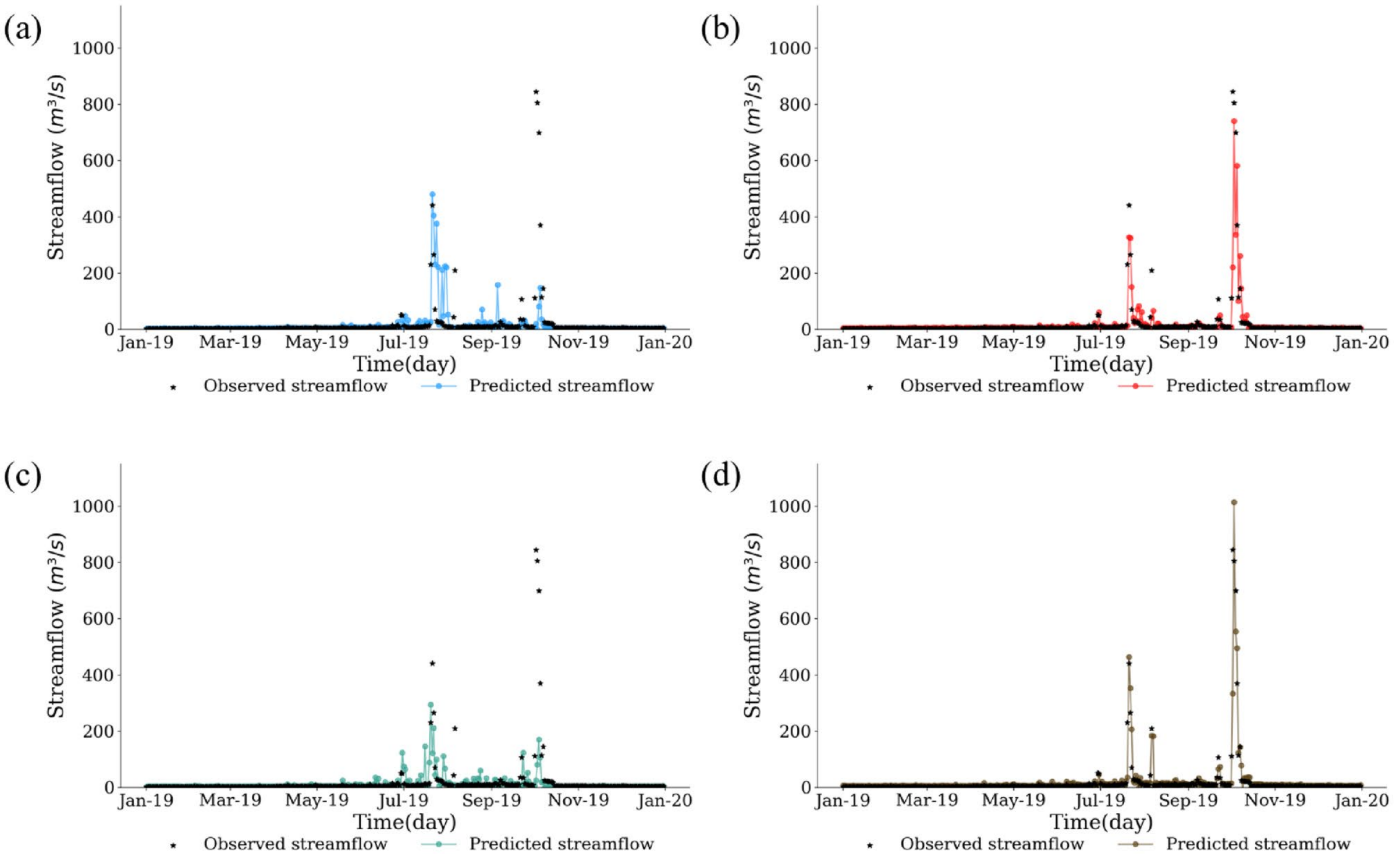
Comparisons between observed and predicted streamflow during the year of 2019 for (**a**) scenario #1, (**b**) scenario #2, (**c**) scenario #3, and (**d**) scenario #4 at the streamflow station (4,008,660).

We visually compared the simulated and observed values for the streamflow station (4008660) with the greatest improvement (Figs. 7, 8, and 9). In the scatter plot (Fig. 7), all scenarios showed decent predictions during the training period, but Scenarios #1 and #3 without the dam data tended not to capture the actual peak streamflow during the test period. In contrast, Scenarios #2 and #4 with dam/weir operational data captured the peak observed values well during the test period (Fig. 7).

Figures 8 and 9 show the time-series comparisons between observed and simulated values. Like Fig. 7, the results in Fig. 8 show that all scenarios well captured the actual streamflow patterns for the training period (Fig. 8), and the predictions for the test period differed depending on the inclusion of dam data. For example, Scenarios #1 and #3 without dam data showed a noticeable discrepancy between the simulated and observed peak values. However, Scenarios #2 and #4 with dam data relatively well predicted the observed peak streamflow. As a result, the streamflow time series during the year of 2019 were analyzed for better comparisons of streamflow prediction based on dam data, as shown in Fig. 9. Focusing on October at the year of 2019, which was affected by the typhoon, the greatest recorded streamflow period was 845.04 mm. However, Scenario #1 and Scenario #3 without the dam data predicted only 149.15 mm and 171.62 mm, respectively, less than half of the observed value. In contrast, Scenario #2 and Scenario #4 with the dam data predicted 581.75 mm and 1013.88 mm, respectively. These time series results supported the LSTM model well depicted peak streamflow when including dam/weir data.

Overall, the performance of LSTM models was lower during the test period compared to during the train period (Fig. 7). The LSTM model is the deep learning model developed to handle time-series data. The pattern of time-series data is trained by the model during the train period, and the trained model is applied into the test period data. The average summertime precipitation during the train period was 248 mm lower than one during the test period (Table S11 of the Supplementary Material). The great difference of summertime precipitation between the train and test periods likely reduced the LSTM performance during the test period relative to during the train period. Therefore, the trained LSTM model performed well in the year of 2019 than in the year of 2020 because the 2019 summer had fewer precipitation than the 2020 summer (Fig. 8). In the future research, weather conditions should be carefully considered to maintain the predictive power of the LSTM model during the train and test periods.

**Figure 10:**
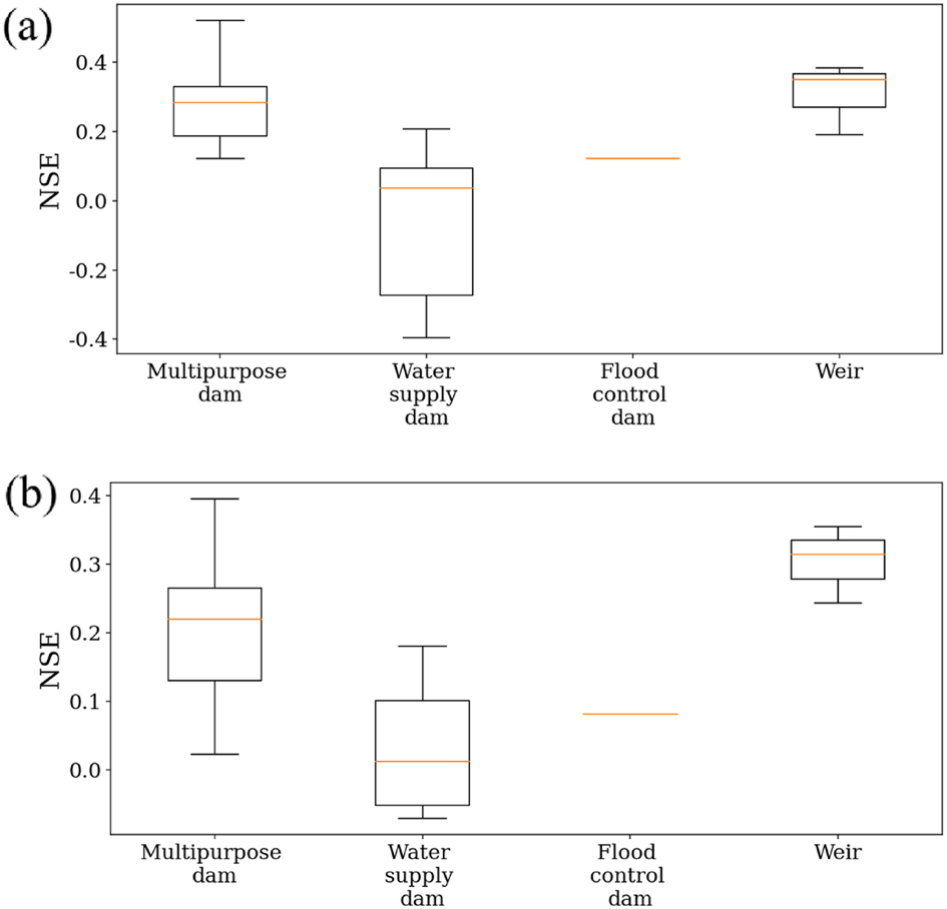
Boxplot of performance differences between (**a**) scenario #1 and scenario #2, and between (**b**) scenario #3 and scenario #4, depending on dam/weir types.

We investigated the LSTM performance according to the dam/weir type (multipurpose dams, water supply dams, flood control dams, and weirs, Fig. 10). Discharge from the dam/weir was controlled according to the intended use. Multipurpose dams are designed for stable water supply and hydropower generation and thus those dams continuously release water, exerting a steady influence on the downstream area. Water supply dams intermittently discharge water for flood control. Flood control dams are built to prevent flood damage in downstream areas in the event of sudden and severe flooding in upstream areas, and dam gates are adjusted during flooding events. Weirs are used for stable flow management, small-scale hydropower generation, and water supply for domestic and industrial use. They continuously release water to maintain an appropriate water level and generate hydropower. Regarding operational characteristics, multipurpose dams and weirs significantly and steadily impact downstream waters.

In compliance with the operational characteristics, the performance difference between Scenarios #1 and #2 varied by the dam type and weir, with median NSE values of 0.283, 0.037, 0.122, and 0.288 for multipurpose dams, water supply dams, flood control dams, and weirs, respectively (Fig. 10). The median values of NSE difference between Scenarios #3 and #4 were 0.220 (multipurpose dams), 0.012 (water supply dams), 0.081 (flood control dams), and 0.279 (weir). Multipurpose dams and weirs showed an improvement in NSE values of 0.2~, whereas the flood control dam showed an improvement in NSE values of ~ 0.1. However, in the case of water-supply dams, performance decreased or recorded minimal performance improvement due to dam operational characteristics. The performance differences among the HR, YSR, and NR basin were also affected by the operational characteristics. Most streamflow stations (85% >) in the HR and YSR basins were affected by multipurpose dams and weirs while only 60% of streamflow stations in the NR basin were downstream of multipurpose dams and weirs (Table 2). Therefore, when dam/weir operational data were added to predict streamflow using deep learning models, understanding of their operational characteristics.

**Table 2:** The number (percent) of streamflow stations affected by different dam/weir for the individual three basins.

Type	HR	NR	YSR
Multipurpose dam	6 (75%)	4 (40%)	6 (85.7%)
Water supply dam	0 (0%)	4 (40%)	1 (14.3%)
Flood control dam	1 (12.5%)	0 (0%)	0 (0%)
Weir	1 (12.5%)	2 (20%)	0 (0%)
Total	8 (100%)	10 (100%)	7 (100%)

HR, NR, and YSR represent the Han River, Nakdong River, and Youngsan/Seomjin River, respectively. The numbers outside and inside the parenthesis indicate the number and percentage of streamflow stations, respectively.

### Implications and limitations

The findings from this study have practical implications for the water resources sector, as more accurate streamflow predictions can support better decision-making in water resources management, including flood prevention, water allocation, and ecosystem preservation. By incorporating dam/weir operational data, the proposed LSTM model can help water resource managers to make more informed decisions on dam/weir operation, reducing the risk of water-related disasters and enhancing the sustainability of water resources.

This study demonstrated the benefits of incorporating dam/weir operational data into the LSTM model for improved streamflow predictions. While process-based models were often reported to provide accurate predictions on streamflow^55^, there are several advantages on using the deep learning (DL) models over process-based models. Firstly, DL models can capture complex nonlinear relationships between input and output variables that may not be simulated by traditional process-based approaches. This enables more accurate streamflow predictions even in situations where the underlying processes are not entirely understood or are too complex to be represented in a process-based model^56^. Secondly, DL models are computationally efficient and can be used for real-time predictions, which is crucial for effective water resources management in response to rapid changing conditions^57^. Lastly, DL models often require less domain-specific expertise and extensive data compared to process-based models. Process-based models typically necessitate a deep understanding of the underlying physical processes and require substantial data for calibration and validation. In contrast, DL models can be more easily applied across various situations without the need for extensive domain knowledge and can be trained effectively with relatively less data^58^.

The LSTM model has been frequently used to prevent natural disasters. Poornima and Pushpalatha^59^ applied LSTM to enhance rainfall prediction models, aiming to support decision-making processes for disaster prevention. Cho et al.^60^ used LSTM and GRU to improve flood predictions for preventing economic and human loss. Le et al.^61^ designed a model for flood prevention, utilizing LSTM to predict the rapidly changing downstream flow due to discharge from upstream hydropower reservoirs^62^. These studies highlighted LSTM as an effective tool in disaster prediction. As shown in our results, LSTM's performance for the test period was highly dependent on the data patterns during the train period (Fig. 7). The data patterns during the train period were substantially different from those during the test period in this study and thus such discrepancies led to decreased NSE and increased RMSE during the test period relative to those during the train period (Fig. 7). To make full use of LSTM for natural disasters including flooding, the train period should be long-term and include multiple natural disaster events for accurate predictions during the train period. The high dependence of LSTM on the train-period data should be carefully considered for future studies to make accurate predictions.

Following previous studies, this study evaluated the LSTM prediction performance for streamflow using NSE^19^. Since this static (NSE) has been most used to assess the streamflow prediction performance using process-based models^63^ or deep learning models^64,65^, the use of NSE could allow to compare similar previous studies. The focus of this study was to recommend suitable input data for streamflow prediction using the LSTM model in the regions with strong climatic seasonality and prevalent dams/weirs. Therefore, the NSE-based assessment could provide insight on future similar studies.

For future research, it is recommended to explore the influence of various human activities, such as agricultural practices and water treatment facilities, on streamflow predictions. This could be achieved by incorporating diverse datasets that capture the effects of these activities on streamflow Understanding the limitations and possible reasons for these failures will help in refining the model and improving its performance in predicting extreme streamflow events.

## Conclusion

In this study, four scenarios were compared and evaluated to quantify how dam/weir operational data affect streamflow prediction performance. The results showed that the LSTM performance was improved by incorporating the dam/weir operational data. When comparing the LSTM models with and without dam/weir operational data, the NSE values were improved by 0.182–0.206 and the RMSE values were reduced by 78.27–79.6. In particular, the LSTM model with dam/weir operational data outperformed the LSTM model without data on peak streamflow. However, the LSTM model with the dam/weir operational data showed a varying degree of performances. The frequency and amount of water discharged from the dams/weirs downstream differed. When the dam/weir operational data were added to the LSTM model, the degree of prediction improvement tended to increase with dams/weirs that frequently released water. Overall, the LSTM model with weather and dam/weir operational data represented a better prediction performance relative to the model with only weather data. However, not all regions may benefit equally from the addition of dam/weir operational data due to these variations in operational characteristics and their impact on streamflow predictions. Therefore, the dam/weir operational data should be carefully incorporated, considering the dam/weir operational characteristics.

## Supplementary Information

Supplementary Information.

## Data Availability

The datasets used and/or analyzed in this study are available from the corresponding author upon reasonable request.
