# Supplementary material for: Assessing the impacts of dam/weir operation on streamflow predictions using LSTM across South Korea
Source: Sci Rep. 2023 Jun 8;13:9296. doi: 10.1038/s41598-023-36439-z (PMC10250378; doi:10.1038/s41598-023-36439-z)
Supplement: Supplementary file 1 — Supplementary Information. [file 41598_2023_36439_MOESM1_ESM.docx]

**Supplementary material for:**

**Assessing the impacts of dam and weir operation on streamflow predictions using LSTM across South Korea**

Corresponding author: Sangchul Lee ([sangchul.lee84@gmail.com & slee2020@uos.ac.kr)](mailto:sangchul.lee84@gmail.com%20&%20slee2020@uos.ac.kr))

Contents of this file

Tables S1 – S11.

Figures S1, S2.

**Table S1.** The detailed information of weather stations

| Num. | ASOS number | Basin | Latitude | Longitude | Count | Number of missing values | | | | Precipitation (mm/s) | | | Minimum temperature (℃) | | | Maximum temperature (℃) | | | Relative humidity (%) | | |
| --- | --- | --- | --- | --- | --- | --- | --- | --- | --- | --- | --- | --- | --- | --- | --- | --- | --- | --- | --- | --- | --- |
|  |  |  |  |  |  | Precipitation | Minimum temperature | Maximum temperature | Relative humidity | Mean | Min | Max | Mean | Min | Max | Mean | Min | Max | Mean | Min | Max |
| 1 | 98 | HR | 37.9019 | 127.0607 | 4018 | 0 | 2 | 1 | 3 | 3.8 | 0 | 449.5 | 6.6 | -20.7 | 27.8 | 18.1 | -10.0 | 38.7 | 64.8 | 18.9 | 99.8 |
| 2 | 101 | HR | 37.9026 | 127.7357 | 4018 | 0 | 0 | 0 | 1 | 3.6 | 0 | 262.5 | 6.6 | -23.1 | 27.6 | 17.5 | -9.3 | 39.5 | 69.8 | 26.4 | 100.0 |
| 3 | 108 | HR | 37.5714 | 126.9658 | 4018 | 0 | 0 | 1 | 0 | 3.7 | 0 | 301.5 | 9.0 | -18.0 | 30.3 | 17.7 | -10.7 | 39.6 | 59.7 | 17.9 | 99.8 |
| 4 | 127 | HR | 36.9705 | 12.79525 | 4018 | 0 | 0 | 1 | 0 | 3.7 | 0 | 301.5 | 6.7 | -21.7 | 28.5 | 17.7 | -10.7 | 39.6 | 59.7 | 17.9 | 99.8 |
| 5 | 136 | NR | 36.5729 | 128.7073 | 3452 | 0 | 0 | 0 | 0 | 3.7 | 0 | 301.5 | 7.4 | -20.4 | 26.4 | 17.7 | -10.7 | 39.6 | 59.7 | 17.9 | 99.8 |
| 6 | 143 | NR | 35.8780 | 128.6530 | 3097 | 0 | 0 | 1 | 1 | 3.2 | 0 | 167.0 | 10.1 | -13.9 | 28.6 | 18.1 | -9.2 | 40.0 | 65.9 | 22.5 | 98.5 |
| 7 | 152 | NR | 35.5824 | 129.3347 | 4018 | 0 | 0 | 0 | 0 | 2.7 | 0 | 125.5 | 10.3 | -13.5 | 28.8 | 18.9 | -7.7 | 38.9 | 64.8 | 22.1 | 100.0 |
| 8 | 156 | YSR | 35.1729 | 126.8916 | 4018 | 0 | 0 | 0 | 0 | 3.0 | 0 | 168.2 | 10.2 | -11.7 | 28.0 | 20.2 | -7.6 | 39.2 | 60.4 | 18.6 | 98.3 |
| 9 | 202 | HR | 37.4886 | 127.4945 | 4018 | 0 | 0 | 0 | 16 | 3.5 | 0 | 266.0 | 7.1 | -21.7 | 27.6 | 19.4 | -4.9 | 38.8 | 65.2 | 16.8 | 99.8 |
| 10 | 203 | HR | 37.2640 | 127.4842 | 3097 | 0 | 0 | 0 | 4 | 3.5 | 0 | 266.0 | 6.7 | -22.5 | 27.0 | 19.4 | -4.9 | 38.8 | 65.2 | 16.8 | 99.8 |
| 11 | 247 | YSR | 35.4213 | 127.3965 | 4018 | 0 | 1 | 0 | 7 | 3.8 | 0 | 259.5 | 7.4 | -18.4 | 26.6 | 19.7 | -7.7 | 38.5 | 68.7 | 23.9 | 99.0 |
| 12 | 254 | YSR | 35.3713 | 127.1286 | 4018 | 0 | 2 | 2 | 17 | 3.7 | 0 | 230.0 | 7.6 | -17.7 | 26.3 | 18.1 | -9.9 | 40.1 | 67.4 | 15.5 | 100.0 |
| 13 | 255 | NR | 35.2266 | 128.6726 | 3097 | 0 | 1 | 1 | 10 | 3.2 | 0 | 202.0 | 10.4 | -11.7 | 27.6 | 18.5 | -9.4 | 39.4 | 65.4 | 15.6 | 100.0 |
| 14 | 273 | NR | 36.6273 | 128.1488 | 3452 | 0 | 0 | 0 | 5 | 3.7 | 0 | 289.4 | 7.3 | -17.0 | 26.2 | 18.9 | -8.2 | 37.5 | 68.7 | 29.4 | 100.0 |
| 15 | 278 | NR | 36.3561 | 128.6886 | 2374 | 0 | 0 | 0 | 1 | 3.7 | 0 | 289.4 | 6.1 | -19.3 | 26.8 | 18.9 | -8.2 | 37.5 | 68.7 | 29.4 | 100.0 |
| 16 | 281 | NR | 35.9774 | 128.9514 | 4018 | 0 | 0 | 1 | 5 | 3.7 | 0 | 289.4 | 7.6 | -15.7 | 28.1 | 18.9 | -8.2 | 37.5 | 68.7 | 29.4 | 100.0 |

**Table S2** The detailed information of streamflow stations

| Num. | Station code | Basin | Latitude | Longitude | Count | Number of missing values  (Streamflow) | Streamflow (m^3^/s) | | |
| --- | --- | --- | --- | --- | --- | --- | --- | --- | --- |
|  |  |  |  |  |  |  | Mean | Min | Max |
| 1 | 1005640 | HR | 37.0761 | 127.8811 | 4018 | 0 | 190.9 | 17.5 | 3770.0 |
| 2 | 1007635 | HR | 37.2961 | 127.6475 | 3097 | 0 | 200.6 | 11.1 | 5372.5 |
| 3 | 1013645 | HR | 37.8175 | 127.6336 | 4018 | 0 | 153.5 | 0.9 | 7584.9 |
| 4 | 1018610 | HR | 37.5475 | 127.2391 | 4018 | 12 | 428.2 | 14.6 | 19589.7 |
| 5 | 1018662 | HR | 37.5230 | 127.0625 | 4018 | 78 | 647.9 | 243.3 | 9306.0 |
| 6 | 1018683 | HR | 37.5144 | 126.9566 | 4018 | 27 | 482.5 | -16.2 | 20649.6 |
| 7 | 1019630 | HR | 37.5986 | 126.8105 | 4018 | 70 | 937.0 | 223.0 | 31099.8 |
| 8 | 1023660 | HR | 37.9847 | 126.9188 | 4018 | 37 | 159.2 | 4.2 | 11849.4 |
| 9 | 2002685 | NR | 36.4316 | 128.9108 | 2374 | 19 | 5.7 | 0.0 | 454.5 |
| 10 | 2004640 | NR | 36.7150 | 128.6008 | 3452 | 2 | 13.1 | 0.8 | 546.4 |
| 11 | 2007620 | NR | 36.5425 | 128.2613 | 3452 | 17 | 10.4 | 0.1 | 1225.0 |
| 12 | 2008650 | NR | 36.1938 | 128.5641 | 3889 | 77 | 8.6 | 0.0 | 674.6 |
| 13 | 2012625 | NR | 35.9786 | 128.9683 | 4018 | 31 | 4.3 | 0.0 | 255.3 |
| 14 | 2012640 | NR | 35.9305 | 128.8755 | 4018 | 12 | 13.7 | 1.1 | 1083.2 |
| 15 | 2014640 | NR | 35.7527 | 128.3869 | 3097 | 21 | 189.2 | 11.4 | 7432.4 |
| 16 | 2020615 | NR | 35.3833 | 128.4836 | 3097 | 0 | 328.1 | 1.0 | 11996.9 |
| 17 | 2201653 | NR | 35.5613 | 129.2486 | 4018 | 29 | 5.8 | 0.0 | 800.9 |
| 18 | 2201660 | NR | 35.5538 | 129.2752 | 4018 | 25 | 10.1 | 0.3 | 909.2 |
| 19 | 4002690 | YSR | 35.4236 | 127.2158 | 4018 | 17 | 9.1 | 0.1 | 1709.7 |
| 20 | 4004660 | YSR | 35.3313 | 127.2163 | 4018 | 18 | 28.1 | 1.4 | 3336.2 |
| 21 | 4004690 | YSR | 35.3113 | 127.2958 | 4018 | 0 | 28.9 | 0.8 | 4906.5 |
| 22 | 4006660 | YSR | 35.2947 | 127.3297 | 4018 | 15 | 40.6 | 0.0 | 7102.5 |
| 23 | 4008660 | YSR | 35.1277 | 127.2988 | 4018 | 0 | 15.4 | 1.6 | 1005.8 |
| 24 | 4008670 | YSR | 35.1602 | 127.3466 | 4018 | 17 | 18.1 | 1.7 | 1690.9 |
| 25 | 5002690 | YSR | 35.1341 | 126.7850 | 4018 | 0 | 11.6 | 0.1 | 2104.8 |

**Table S3.** The detailed information of dam/weir

| Num. | Name | Type | Basin | Release start date | Latitude | Longitude | Count | Number of missing values | | Inflow (m^3^/s) | | | Outflow (m^3^/s) | | |
| --- | --- | --- | --- | --- | --- | --- | --- | --- | --- | --- | --- | --- | --- | --- | --- |
|  |  |  |  |  |  |  |  | Inflow | Outflow | Mean | Min | Max | Mean | Min | Max |
| 1 | Paldang | Multipurpose dam | HR | 2010-01-01 | 37.5262 | 127.2790 | 4018 | 15 | 15 | 467.3 | 0.0 | 15922.5 | 469.7 | 0.0 | 15975.4 |
| 2 | Gunnam | Flood control dam | HR | 2010-01-01 | 38.1043 | 127.0179 | 4018 | 0 | 0 | 83.7 | 1.4 | 9779.9 | 83.8 | 1.1 | 9198.1 |
| 3 | Uiam | Multipurpose dam | HR | 2010-01-01 | 37.8361 | 127.6758 | 4018 | 13 | 13 | 141.4 | 0.0 | 9948.8 | 141.7 | 0.0 | 9903.6 |
| 4 | Chungju | Multipurpose dam | HR | 2010-01-01 | 37.0060 | 127.9925 | 4018 | 0 | 0 | 137.0 | 0.0 | 3960.4 | 137.8 | 0.0 | 5059.9 |
| 5 | Seomjingang | Multipurpose dam | YSR | 2010-01-01 | 35.5409 | 127.1100 | 4018 | 0 | 0 | 18.9 | 1.1 | 1397.0 | 19.2 | 0.0 | 2001.0 |
| 6 | Pyeongrim | Water supply dam | YSR | 2010-01-01 | 35.2877 | 126.6853 | 4018 | 0 | 0 | 0.5 | 0.0 | 38.8 | 0.5 | 0.0 | 39.3 |
| 7 | Juam | Multipurpose dam | YSR | 2010-01-01 | 35.0639 | 127.2378 | 4018 | 0 | 0 | 21.5 | 6.3 | 1008.2 | 21.8 | 0.0 | 1716.2 |
| 8 | Yeongju | Multipurpose dam | NR | 2011-07-21 | 36.7221 | 128.6562 | 3452 | 0 | 0 | 6.1 | 0.0 | 321.6 | 6.5 | 0.0 | 383.4 |
| 9 | Seongdeok | Multipurpose dam | NR | 2014-07-03 | 36.2409 | 128.9638 | 2374 | 0 | 0 | 0.6 | 0.0 | 22.1 | 0.7 | 0.0 | 63.9 |
| 10 | Yeongcheon | Water supply dam | NR | 2010-01-01 | 36.0639 | 129.0138 | 4018 | 0 | 0 | 7.3 | 0.4 | 154.0 | 7.4 | 0.0 | 313.2 |
| 11 | Gunwi | Multipurpose dam | NR | 2010-05-10 | 36.1200 | 128.7954 | 3889 | 0 | 0 | 1.1 | 0.0 | 51.9 | 1.2 | 0.0 | 105.2 |
| 12 | Sayeon | Water supply dam | NR | 2010-01-01 | 35.5803 | 129.1940 | 4018 | 0 | 0 | 2.3 | 0.0 | 270.0 | 2.3 | 0.0 | 237.8 |
| 13 | Gangcheon | Weir | HR | 2012-07-10 | 37.2791 | 127.6846 | 3097 | 0 | 0 | 187.4 | 21.7 | 5208.6 | 187.4 | 21.7 | 5293.0 |
| 14 | Gangjeong-Goryeong | Weir | NR | 2012-07-10 | 35.8406 | 128.4603 | 3097 | 0 | 0 | 164.3 | 0.0 | 6681.4 | 164.3 | 0.0 | 6322.8 |
| 15 | Hapcheong-Changnyeong | Weir | NR | 2012-07-10 | 35.5948 | 128.3568 | 3097 | 0 | 0 | 219.9 | 0.0 | 11908.1 | 219.7 | 0.0 | 11481.7 |

**Table S4.** Matching of Streamflow, Weather stations and Dam/Weir.

| Num. | Streamflow station code | Weather station code | Name of Dam/Weir |
| --- | --- | --- | --- |
| 1 | 1005640 | 127 | Chungju |
| 2 | 1007635 | 203 | Gangcheon |
| 3 | 1013645 | 101 | Uiam |
| 4 | 1018610 | 202 | Paldang |
| 5 | 1018662 | 108 | Paldang |
| 6 | 1018683 | 108 | Paldang |
| 7 | 1019630 | 108 | Paldang |
| 8 | 1023660 | 98 | Gunnam |
| 9 | 2002685 | 278 | Seongdeok |
| 10 | 2004640 | 136 | Yeongju |
| 11 | 2007620 | 273 | Yeongju |
| 12 | 2008650 | 278 | Gunwi |
| 13 | 2012625 | 281 | Yeongcheon |
| 14 | 2012640 | 281 | Yeongcheon |
| 15 | 2014640 | 143 | Gangjeong-Goryeong |
| 16 | 2020615 | 255 | Hapcheong-Changnyeong |
| 17 | 2201653 | 152 | Sayeon |
| 18 | 2201660 | 152 | Sayeon |
| 19 | 4002690 | 254 | Seomjingang |
| 20 | 4004660 | 254 | Seomjingang |
| 21 | 4004690 | 247 | Seomjingang |
| 22 | 4006660 | 247 | Seomjingang |
| 23 | 4008660 | 254 | Juam |
| 24 | 4008670 | 247 | Juam |
| 25 | 5002690 | 156 | Pyeongrim |

**Table S5.** The optimal hyperparameter values for Scenarios #1 and #2

| Hyperparameters | Scenario #1 | Scenario #2 |
| --- | --- | --- |
| Window size | 7 | 7 |
| Number of nodes (1^st^ LSTM layer) | 128 | 128 |
| Number of nodes (2^nd^ LSTM layer) | 64 | 64 |
| Number of nodes (1^st^ Dense layer) | 128 | 128 |
| Number of nodes (2^nd^ Dense layer) | 64 | 64 |
| Dropout | 0.5 | 0.5 |
| Learning Rate | 0.001 | 0.001 |
| Batch Size | 128 | 128 |
| Epochs | 500 | 500 |

**Table S6.** Hyperparameter range for Bayesian Optimization

| Hyperparameters | Range |
| --- | --- |
| Window size | 1, 2, 3, 4, 5, 6, 7 |
| Number of nodes (1^st^ LSTM layer) | 16, 32, 64, 128, 256, 512 |
| Number of nodes (2^nd^ LSTM layer) | 16, 32, 64, 128, 256, 512 |
| Number of nodes (1^st^ Dense layer) | 16, 32, 64, 128, 256, 512 |
| Number of nodes (2^nd^ Dense layer) | 16, 32, 64, 128, 256, 512 |
| Dropout | 0.1, 0.2, 0.3, 0.4, 0.5 |
| Learning Rate | 0.1, 0.01, 0.001, 0.0001 |
| Batch Size | 32, 64, 128, 256 |
| Epochs | 100, 200, 300, 400, 500 |

**Table S7.** The optimal hyperparameter values for Scenario #3

| Station code | Window size | Number of nodes (1^st^ LSTM layer) | Number of nodes (2^nd^ LSTM layer) | Number of nodes (1^st^ Dense layer) | Number of nodes (2^nd^ Dense layer) | Dropout rate | Learning Rate | Batch Size | Epochs |
| --- | --- | --- | --- | --- | --- | --- | --- | --- | --- |
| 1005640 | 7 | 128 | 32 | 16 | 32 | 0.4 | 0.001 | 32 | 400 |
| 1007635 | 7 | 16 | 128 | 256 | 256 | 0.2 | 0.001 | 32 | 400 |
| 1013645 | 7 | 512 | 256 | 256 | 512 | 0.5 | 0.001 | 32 | 200 |
| 1018610 | 6 | 256 | 256 | 512 | 256 | 0.5 | 0.01 | 32 | 500 |
| 1018662 | 6 | 32 | 256 | 256 | 128 | 0.3 | 0.001 | 64 | 300 |
| 1018683 | 7 | 64 | 64 | 256 | 256 | 0.4 | 0.001 | 32 | 300 |
| 1019630 | 7 | 128 | 32 | 512 | 256 | 0.5 | 0.01 | 32 | 500 |
| 1023660 | 3 | 64 | 128 | 512 | 64 | 0.2 | 0.01 | 64 | 500 |
| 2002685 | 2 | 256 | 256 | 512 | 16 | 0.1 | 0.001 | 64 | 500 |
| 2004640 | 7 | 16 | 16 | 512 | 512 | 0.2 | 0.01 | 64 | 500 |
| 2007620 | 6 | 512 | 256 | 512 | 64 | 0.5 | 0.01 | 256 | 500 |
| 2008650 | 1 | 256 | 16 | 64 | 16 | 0.1 | 0.01 | 32 | 500 |
| 2012625 | 2 | 32 | 256 | 16 | 32 | 0.3 | 0.001 | 32 | 400 |
| 2012640 | 2 | 512 | 64 | 512 | 32 | 0.2 | 0.01 | 32 | 300 |
| 2014640 | 7 | 256 | 128 | 64 | 512 | 0.2 | 0.001 | 32 | 400 |
| 2020615 | 4 | 256 | 128 | 64 | 256 | 0.2 | 0.001 | 32 | 500 |
| 2201653 | 2 | 64 | 256 | 32 | 32 | 0.3 | 0.001 | 32 | 500 |
| 2201660 | 7 | 256 | 512 | 64 | 64 | 0.1 | 0.01 | 256 | 200 |
| 4002690 | 2 | 256 | 64 | 32 | 32 | 0.3 | 0.001 | 32 | 500 |
| 4004660 | 3 | 128 | 512 | 16 | 64 | 0.3 | 0.01 | 256 | 400 |
| 4004690 | 3 | 256 | 256 | 64 | 16 | 0.1 | 0.01 | 256 | 400 |
| 4006660 | 3 | 64 | 128 | 32 | 128 | 0.3 | 0.01 | 128 | 500 |
| 4008660 | 7 | 64 | 512 | 256 | 512 | 0.1 | 0.001 | 32 | 300 |
| 4008670 | 7 | 64 | 64 | 64 | 256 | 0.4 | 0.001 | 64 | 500 |
| 5002690 | 4 | 32 | 16 | 512 | 128 | 0.4 | 0.01 | 64 | 500 |

**Table S8.** The optimal hyperparameter values for Scenario #4

| Station code | Window size | Number of nodes (1^st^ LSTM layer) | Number of nodes (2^nd^ LSTM layer) | Number of nodes (1^st^ Dense layer) | Number of nodes (2^nd^ Dense layer) | Dropout rate | Learning Rate | Batch Size | Epochs |
| --- | --- | --- | --- | --- | --- | --- | --- | --- | --- |
| 1005640 | 6 | 32 | 16 | 256 | 128 | 0.4 | 0.001 | 64 | 300 |
| 1007635 | 1 | 32 | 256 | 512 | 64 | 0.1 | 0.0001 | 128 | 200 |
| 1013645 | 6 | 16 | 256 | 64 | 512 | 0.3 | 0.001 | 64 | 400 |
| 1018610 | 5 | 16 | 256 | 512 | 256 | 0.1 | 0.01 | 128 | 400 |
| 1018662 | 1 | 16 | 16 | 128 | 32 | 0.3 | 0.01 | 32 | 400 |
| 1018683 | 1 | 512 | 512 | 128 | 128 | 0.4 | 0.01 | 32 | 500 |
| 1019630 | 6 | 128 | 512 | 64 | 128 | 0.3 | 0.0001 | 64 | 500 |
| 1023660 | 1 | 32 | 16 | 128 | 32 | 0.2 | 0.01 | 32 | 500 |
| 2002685 | 1 | 32 | 256 | 16 | 128 | 0.1 | 0.1 | 128 | 400 |
| 2004640 | 3 | 64 | 64 | 16 | 256 | 0.1 | 0.01 | 64 | 300 |
| 2007620 | 3 | 16 | 128 | 16 | 256 | 0.1 | 0.1 | 128 | 500 |
| 2008650 | 1 | 256 | 16 | 16 | 32 | 0.5 | 0.0001 | 32 | 100 |
| 2012625 | 7 | 256 | 16 | 32 | 32 | 0.5 | 0.001 | 256 | 500 |
| 2012640 | 6 | 32 | 16 | 16 | 128 | 0.3 | 0.001 | 32 | 300 |
| 2014640 | 3 | 32 | 256 | 128 | 128 | 0.5 | 0.001 | 64 | 400 |
| 2020615 | 4 | 256 | 256 | 64 | 16 | 0.5 | 0.01 | 64 | 500 |
| 2201653 | 2 | 64 | 128 | 32 | 128 | 0.2 | 0.001 | 64 | 300 |
| 2201660 | 7 | 64 | 128 | 16 | 128 | 0.4 | 0.001 | 32 | 400 |
| 4002690 | 2 | 32 | 16 | 64 | 64 | 0.1 | 0.1 | 256 | 400 |
| 4004660 | 1 | 256 | 16 | 32 | 16 | 0.3 | 0.01 | 32 | 400 |
| 4004690 | 6 | 128 | 256 | 128 | 512 | 0.3 | 0.01 | 128 | 300 |
| 4006660 | 2 | 64 | 128 | 256 | 512 | 0.2 | 0.1 | 32 | 300 |
| 4008660 | 4 | 32 | 512 | 128 | 128 | 0.1 | 0.001 | 32 | 300 |
| 4008670 | 1 | 16 | 64 | 128 | 64 | 0.4 | 0.0001 | 128 | 200 |
| 5002690 | 2 | 16 | 64 | 512 | 16 | 0.4 | 0.01 | 64 | 500 |

| Num. | Station code | Scenario #1 | Scenario #2 | Scenario #3 | Scenario #4 |
| --- | --- | --- | --- | --- | --- |
| 1 | 1005640 | 0.382 (0.948) | 0.868 (0.980) | 0.530 (0.641) | 0.901 (0.910) |
| 2 | 1007635 | 0.277 (0.910) | 0.627 (0.983) | 0.436 (0.633) | 0.791 (0.711) |
| 3 | 1013645 | 0.469 (0.955) | 0.796 (0.976) | 0.658 (0.871) | 0.796 (0.934) |
| 4 | 1018610 | 0.501 (0.972) | 0.675 (0.988) | 0.593 (0.783) | 0.859 (0.932) |
| 5 | 1018662 | 0.539 (0.968) | 0.799 (0.972) | 0.597 (0.787) | 0.889 (0.804) |
| 6 | 1018683 | 0.418 (0.975) | 0.756 (0.823) | 0.596 (0.958) | 0.829 (0.808) |
| 7 | 1019630 | 0.350 (0.953) | 0.667 (0.939) | 0.503 (0.926) | 0.760 (0.814) |
| 8 | 1023660 | 0.521 (0.975) | 0.643 (0.987) | 0.586 (0.806) | 0.667 (0.994) |
| 9 | 2002685 | 0.132 (0.959) | 0.254 (0.945) | 0.361 (0.612) | 0.466 (0.937) |
| 10 | 2004640 | 0.416 (0.915) | 0.545 (0.944) | 0.458 (0.850) | 0.566 (0.899) |
| 11 | 2007620 | 0.214 (0.967) | 0.339 (0.975) | 0.281 (0.864) | 0.488 (0.836) |
| 12 | 2008650 | -0.064 (0.949) | 0.227 (0.960) | 0.433 (0.588) | 0.456 (0.366) |
| 13 | 2012625 | 0.136 (0.879) | 0.230 (0.854) | 0.365 (0.745) | 0.377 (0.924) |
| 14 | 2012640 | 0.173 (0.931) | 0.380 (0.933) | 0.284 (0.684) | 0.385 (0.939) |
| 15 | 2014640 | 0.405 (0.958) | 0.596 (0.960) | 0.419 (0.988) | 0.733 (0.769) |
| 16 | 2020615 | 0.289 (0.953) | 0.673 (0.959) | 0.456 (0.942) | 0.699 (0.924) |
| 17 | 2201653 | 0.233 (0.942) | -0.040 (0.898) | 0.266 (0.595) | 0.195 (0.617) |
| 18 | 2201660 | 0.262 (0.873) | -0.134 (0.930) | 0.212 (0.266) | 0.160 (0.612) |
| 19 | 4002690 | 0.161 (0.919) | 0.352 (0.955) | 0.363 (0.792) | 0.628 (0.913) |
| 20 | 4004660 | 0.293 (0.972) | 0.637 (0.956) | 0.622 (0.547) | 0.727 (0.492) |
| 21 | 4004690 | 0.162 (0.962) | 0.488 (0.963) | 0.128 (0.429) | 0.335 (0.796) |
| 22 | 4006660 | 0.132 (0.812) | 0.329 (0.964) | 0.307 (0.486) | 0.498 (0.804) |
| 23 | 4008660 | 0.129 (0.983) | 0.650 (0.971) | 0.266 (0.992) | 0.661 (0.813) |
| 24 | 4008670 | 0.239 (0.972) | 0.514 (0.964) | 0.344 (0.661) | 0.578 (0.904) |
| 25 | 5002690 | 0.150 (0.931) | 0.187 (0.948) | 0.182 (0.247) | 0.362 (0.732) |
| Mean | | 0.277 (0.941) | 0.482 (0.949) | 0.410 (0.708) | 0.592 (0.807) |
| Median | | 0.262 (0.953) | 0.545 (0.960) | 0.419 (0.745) | 0.628 (0.814) |
| Min | | -0.064 (0.812) | -0.134 (0.823) | 0.128 (0.247) | 0.160 (0.366) |
| Max | | 0.539 (0.983) | 0.868 (0.988) | 0.658 (0.992) | 0.901 (0.994) |

**Table S9.** NSE values of individual streamflow stations.

Note: The numbers outside and inside the parenthesis indicate the NSE values for test and train periods, respectively.

| Num. | Station code | Scenario #1 | Scenario #2 | Scenario #3 | Scenario #4 |
| --- | --- | --- | --- | --- | --- |
| 1 | 1005640 | 242.94 (66.60) | 112.26 (41.14) | 212.06 (174.14) | 97.11 (87.40) |
| 2 | 1007635 | 432.40 (75.75) | 310.83 (33.29) | 381.86 (152.93) | 232.09 (136.29) |
| 3 | 1013645 | 292.79 (61.77) | 181.42 (45.50) | 234.90 (104.71) | 181.31 (74.98) |
| 4 | 1018610 | 793.95 (156.08) | 640.83 (103.72) | 717.29 (436.61) | 422.34 (244.36) |
| 5 | 1018662 | 628.35 (159.34) | 415.02 (147.86) | 587.81 (409.85) | 308.17 (392.60) |
| 6 | 1018683 | 955.91 (165.72) | 619.10 (441.47) | 796.00 (215.90) | 517.25 (459.77) |
| 7 | 1019630 | 1,118.05 (290.66) | 800.37 (328.97) | 977.27 (364.37) | 677.67 (576.26) |
| 8 | 1023660 | 462.48 (89.56) | 399.40 (64.33) | 429.84 (249.25) | 385.59 (43.21) |
| 9 | 2002685 | 28.09 (3.66) | 26.04 (4.24) | 24.07 (11.24) | 22.04 (4.53) |
| 10 | 2004640 | 32.76 (8.16) | 28.93 (6.62) | 31.58 (10.82) | 28.18 (8.92) |
| 11 | 2007620 | 86.18 (5.39) | 79.02 (4.67) | 82.39 (9.59) | 72.21 (11.93) |
| 12 | 2008650 | 28.14 (7.47) | 23.98 (6.63) | 20.52 (21.96) | 20.06 (26.31) |
| 13 | 2012625 | 13.66 (2.66) | 12.89 (2.92) | 11.68 (3.86) | 11.59 (2.10) |
| 14 | 2012640 | 46.74 (10.61) | 40.48 (10.47) | 43.40 (22.66) | 40.30 (9.97) |
| 15 | 2014640 | 489.05 (65.15) | 402.84 (63.14) | 482.75 (35.18) | 326.93 (153.45) |
| 16 | 2020615 | 818.63 (109.51) | 555.06 (102.47) | 714.64 (122.37) | 532.50 (139.86) |
| 17 | 2201653 | 25.86 (5.52) | 30.11 (7.32) | 25.29 (14.61) | 26.49 (14.21) |
| 18 | 2201660 | 36.00 (9.60) | 44.64 (7.12) | 37.21 (23.04) | 38.41 (16.76) |
| 19 | 4002690 | 58.06 (6.35) | 51.02 (4.76) | 50.52 (10.19) | 38.63 (6.58) |
| 20 | 4004660 | 121.78 (14.28) | 87.23 (17.87) | 88.97 (57.15) | 75.56 (60.55) |
| 21 | 4004690 | 155.06 (19.56) | 121.13 (19.48) | 157.92 (76.11) | 137.80 (45.49) |
| 22 | 4006660 | 230.95 (53.78) | 203.00 (23.49) | 206.01 (89.00) | 175.57 (55.03) |
| 23 | 4008660 | 74.37 (5.96) | 47.17 (7.73) | 68.25 (4.16) | 46.32 (19.57) |
| 24 | 4008670 | 79.68 (9.83) | 63.66 (10.98) | 73.99 (33.91) | 59.25 (18.05) |
| 25 | 5002690 | 63.18 (8.48) | 61.78 (7.41) | 61.79 (28.08) | 54.60 (16.75) |
| Mean | | 292.60 (56.46) | 214.33 (60.54) | 260.72 (107.27) | 181.12 (105.00) |
| Median | | 121.78 (14.28) | 87.23 (17.87) | 88.97 (35.18) | 75.56 (43.21) |
| Min | | 13.66 (2.66) | 12.89 (2.92) | 11.68 (3.86) | 11.59 (2.10) |
| Max | | 1,118.05 (290.66) | 800.37 (441.47) | 977.27 (436.61) | 677.67 (576.26) |

**Table S10.** RMSE values for individual streamflow stations

Note: The numbers outside and inside the parenthesis indicate the NSE values for test and train periods, respectively.

**Table S11.** The amount (mm) of monthly precipitation during the train and test periods

| Month | Train period | | | | | | | | Test period | | |
| --- | --- | --- | --- | --- | --- | --- | --- | --- | --- | --- | --- |
|  | 2010 | 2011 | 2012 | 2013 | 2014 | 2015 | 2016 | 2017 | 2018 | 2019 | 2020 |
| January | 33.05 | 4 | 17.1 | 23.6 | 6.5 | 35.8 | 34.7 | 17.6 | 31.55 | 10.3 | 80.9 |
| February | 126.9 | 71.7 | 9.8 | 56.1 | 6.1 | 19.5 | 50.7 | 47.8 | 21.3 | 39.7 | 63 |
| March | 71.05 | 22.5 | 102.1 | 81.6 | 101.6 | 38.6 | 78.15 | 29.1 | 103.7 | 38.5 | 38.2 |
| April | 112.5 | 122.4 | 82.1 | 88.9 | 87.2 | 142.2 | 201.6 | 65.6 | 181.8 | 87.5 | 55.4 |
| May | 123.6 | 134.3 | 44.2 | 90.3 | 63.5 | 51.1 | 91.0 | 41.8 | 106.9 | 84.8 | 93.6 |
| June | 46.35 | 133.6 | 73.6 | 87.7 | 108.4 | 113.2 | 96.5 | 23.5 | 224.8 | 164.6 | 180.4 |
| July | 434.5 | 337.8 | 401.7 | 410.4 | 239.2 | 154.7 | 388.4 | 343.1 | 103.7 | 334.6 | 560.6 |
| August | 531.2 | 363.9 | 434.3 | 243.6 | 440.5 | 124.5 | 71.7 | 229.5 | 427.4 | 114.2 | 822.1 |
| September | 190.5 | 38.5 | 270.7 | 70.7 | 178.8 | 74.5 | 230.5 | 107.8 | 120.2 | 174.3 | 168.3 |
| October | 50.5 | 51 | 58.7 | 51.4 | 113.7 | 99.9 | 199.2 | 86.7 | 125.6 | 178.2 | 9.5 |
| November | 8.9 | 154.3 | 52.1 | 80 | 98.6 | 126.6 | 21.6 | 3.7 | 51 | 27.4 | 48.1 |
| December | 39.55 | 8.4 | 62.2 | 19.85 | 33.9 | 81.2 | 47.45 | 28.7 | 26.4 | 31.45 | 14.5 |

Note: the shaded rows indicate the summer months.


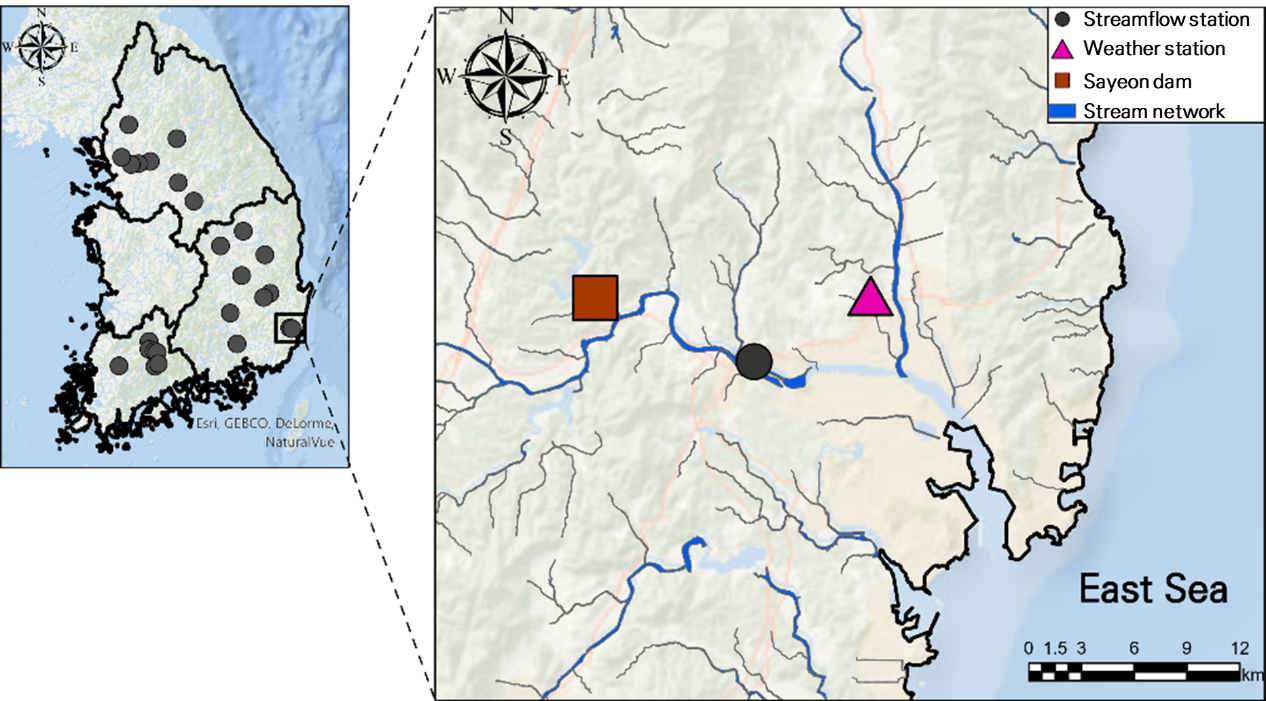


**Fig S1.** Streamflow station located downstream of the Sayeon dam (Ulsan-si Samho Bridge)


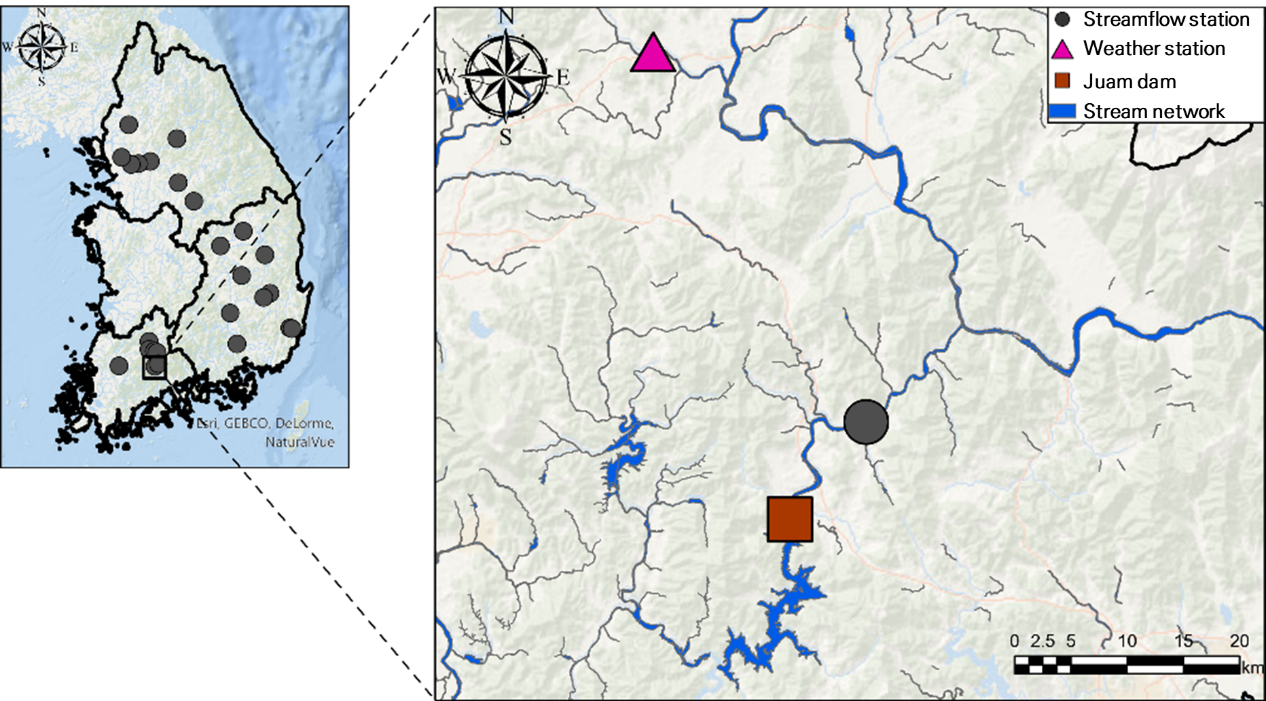


**Fig S2.** The station with the best prediction (Gokseong-gun Moksa-dong 1 Bride)
